# Versatile, Immersive, Creative and Dynamic Virtual 3-D Healthcare Learning Environments: A Review of the Literature

**DOI:** 10.2196/jmir.1051

**Published:** 2008-09-01

**Authors:** Margaret M Hansen

**Affiliations:** ^1^School of NursingUniversity of San FranciscoSan FranciscoCAUSA

**Keywords:** Education, Healthcare, Technology

## Abstract

The author provides a critical overview of three-dimensional (3-D) virtual worlds and “serious gaming” that are currently being developed and used in healthcare professional education and medicine. The relevance of this e-learning innovation for teaching students and professionals is debatable and variables influencing adoption, such as increased knowledge, self-directed learning, and peer collaboration, by academics, healthcare professionals, and business executives are examined while looking at various Web 2.0/3.0 applications. There is a need for more empirical research in order to unearth the pedagogical outcomes and advantages associated with this e-learning technology. A brief description of Roger’s Diffusion of Innovations Theory and Siemens’ Connectivism Theory for today’s learners is presented as potential underlying pedagogical tenets to support the use of virtual 3-D learning environments in higher education and healthcare.

## Introduction

Despite the accelerating momentum of the development, application, and adoption of immersive three-dimensional (3-D) virtual worlds by academics as learning innovations [1,8,12,15,27,28,33,34,35], there are some fundamental questions which remain unanswered. Without doubt, one of the most widely discussed of these is the relevance associated with teaching medical/healthcare professionals [2,3,4]. Similar to most basic issues in education, this question leads to challenges at various levels of thought, and it is beneficial to address while the race to adopt and implement highly engaging Web 3-D virtual worlds is watched in healthcare professional education. At the philosophical and cognitive levels, the value associated with learning presents variables that influence the rate of adoption by academics. The purported beneficial qualities of virtual worlds, such as immersion, role-playing opportunities, simulation, and personal interaction associated with the technology and its influence on formative and summative learning outcomes, requires analysis. Therefore, Roger’s Diffusion of Innovations Theory [5] and Siemens’ Connectivism Theory [6] for today’s learners will serve as theoretical frameworks for this paper. The purpose of this review is to convey knowledge and ideas that have been established concerning the use of 3-D virtual worlds in medical and health professional education to date whilst describing, summarizing, evaluating, and clarifying the current literature.

## Virtual Worlds: Overview

A 3-D virtual world, also known as a Massively Multiplayer Virtual World (MMVW), is an example of a Web 2.0/Web 3-D dynamic computer-based application. According to Jutecht [7], the vague term “Web 2.0” is used for what people see as a second form of the World Wide Web (WWW) architecture and applications that enable social publishing, such as blogs and wikis. A few examples of this interactive Web are podcasts, YouTube, and social networks, such as Facebook, Twitter, and TeeBeeDee. Murray [8] outlines many examples of Web 2.0 applications on the Web. Eysenbach [9], found in Barreto [10], explains:

Web 2.0 is a term which refers to a) improved communication and collaboration between people via social-networking technologies, b) improved communication between separate software applications (“mashups”) via open Web standards for describing and accessing data, and c) improved Web interfaces that mimic the real-time responsiveness of desktop applications within a browser window.

Currently, the most popular virtual world used by the general public is Linden Lab’s Second Life (SL) [11]. It portrays the general qualities of a MMVW which include, but are not limited to, streaming audio/video/TV/YouTube collections, 3-D virtual libraries, virtual tourist attractions and destinations, social interactive venues used by multiple, customized animated characters, a health information island, global preparedness discussions, lectures, conferences, and support groups [12]. Presently, it has 6.5 million virtual residents [13] from over 100 countries. US agencies, such as the Centers for Disease Control and the National Institutes of Health conduct meetings in SL to discuss the educational potential of SL [14]. Kusumoto, Shorrock, Heinrichs, Dev, and Youngblood [15] describe a 3-D virtual world online simulation incorporating a Massively Multiplayer Online Game (MMOG) platform, which trains healthcare professionals for a mass casualty event. This is an example of how a virtual world may offer information about disaster preparedness. Furthermore, virtual medical universities exist all over the world [16]. Therefore, 3-D virtual worlds may include MMOGs, which is one type of a “serious game” including educational goals; however, the authors of the Horizon Report [17] argue virtual worlds are not games and provide, rather, examples of “pure” virtual worlds, which include SL [11], “There” [18], and “Active Worlds”[19]. Many of the programs allow the user to create 3-D virtual worlds, socialize, shop, and participate in an educational universe, whereas MMOGs are considered to be more “goal oriented” and may include a multitude of players engaged in “collaborative” gaming events of a competitive nature within a 3-D environment [13,17].

**Figure 1:**
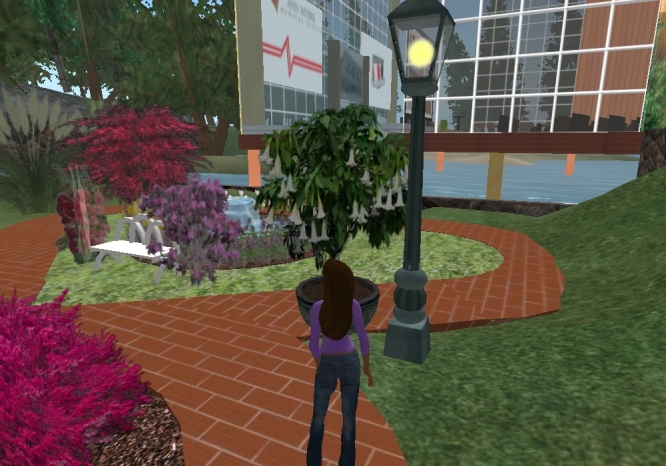
A screenshot of an avatar in Ann Myers Medical Center [20]

Within the virtual 3-D platforms, end users choose a fictitious name from an online menu (eg, the pseudonym of the author is Maggie Waechter) and have the opportunity to create a unique self (eg, human figure, animal or object) known as an “avatar” (Figure 1).

The term “avatar” is an old Sanskrit word portraying a deity which takes on a human shape [21]. These are animated figures the user may navigate to stand, sit, fly, dance, gesture, eat, make love, wind surf, swim, move, walk through doors, open drawers, speak, and “teleport” to various regions and areas within the virtual world via a computer’s keyboard. Furthermore, the player may make and design physical objects and use cash (1000 Lindens = US $4.85) in order to purchase such things as planes, boats, hair, birds, and even body parts. The colourful and creative day-night environment that has a built-in weather system incorporates other Web 2.0 social-networking capacities, such as instant messaging (IM), wikis, users’ ratings, profiles, podcasts, and sharing user-created objects that may be viewed in a virtual world. According to Boulos [12], the current SL program is voice-enabled and provides the player the opportunity to hear other avatar voices based on the avatar’s physical location. For example, if an avatar moves away from another avatar, the avatar’s voice will become fainter and, vice versa, louder as you navigate closer. The New Medium Consortium (NMC) offers a symposium on the evolution of communication and how to use it in SL. The NMC offers media presentations about the creation of movement and emotion in SL educational virtual worlds [22].

Another idea is embedding Wii [23], a gaming software program created by Nintendo, into SL. This combination may offer a plethora of opportunities for all age groups because gaming may motivate end users to log in and have fun while learning. Wii offers a “motion sensitive controller (Wiimote)” and is therefore very suitable for the geriatric population requiring increased range of motion for the hands while building endurance, strength, and coordination [24]. The Wiimote requires body movements very similar to those demonstrated in traditional physical therapy. The patients do rigorous exercises while playing a game [24]. Also, the Wii medical game, “Trauma Center: Second Opinion,” affords players of the game the opportunity to be a surgeon by using the Wiimote. The intuitive nature of the Wiimote allows for experiential learning and the gaming factor may evoke competitiveness, fun, and active learning.

Despite the theme-park atmosphere or paper-doll quality of virtual worlds such as SL, audiences socialize, communicate, build, seek facts, conduct business and participate in other Web 2.0 applications online. Residents in SL may benefit from each other’s participation via networks, which offer dynamic, evolving systems reflecting aspects of the semantic web [25,26]. Researchers make it a point to identify and evaluate budding technologies having an effect on teaching and learning in higher education and forecast a timeframe for potential adoption in education [17]. Virtual worlds are currently being used as educational spaces [1] and continue to grow in popularity on campuses and businesses worldwide. Furthermore, access to versions of virtual worlds on the Web, such as “Croquet,” “Uni-Verse,” and “Multiverse” are predicted within two to three years to be mainstream in education [17].

## Pedagogical Potential

Many authors are expounding the educational and research potential of virtual worlds and MMOGs [12,13,15,16,25,27,28]; however, educational research involving the use and effectiveness of these innovative technologies is in its infancy. More research is necessary regarding the educational outcomes before collaborative encounters in virtual worlds are adopted [28]. Nonetheless, there are reported advantages to having students engage in these emerging technologies [1]. Learners actively interact with content and role play skills associated with their profession. By allowing students time to interact with other avatars (eg, patients, staff members, and other healthcare professionals) in a safe, simulated environment, a decrease in student anxiety, an increase in competency in learning a new skill, and encouragement to cooperate and collaborate, as well as resolve conflicts, is possible. Active learning takes place due to other participants being in the same virtual world and constructing objects to represent ideas that may enhance self-reflection and knowledge [1]. If a gaming component is associated with the 3-D virtual world, the student may be motivated to log in. High quality 3-D entertainment that is freely accessible via Web browsing facilitates engagement opportunities with individuals or groups of people in an authentic manner that illustrates collective intelligence [29].

## Healthcare Professional Education Examples

**Figure 2:**
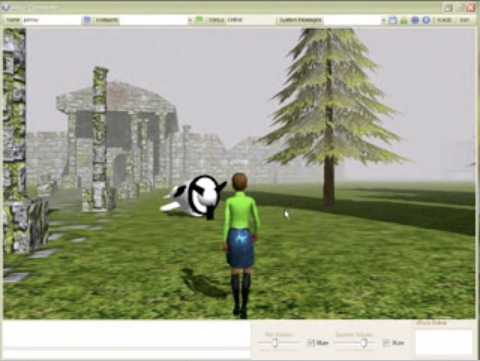
ALIVE classmate tutorial via YouTube video [31]

Another example of a virtual world and MMOG exemplifying global collaboration and fearless creativity is the Advanced Learning and Immersive Virtual Environment (ALIVE) at the University of Southern Queensland (USQ) [30]. The underlying goal of the ALIVE team is to provide educators the opportunity to develop learning content, which is brought to life in 3-D virtual worlds. The ALIVE team provides YouTube video clips on how to use ALIVE Classmate, an online virtual classroom (Figure 2).

The ALIVE DX Editor [32] is a simple-to-use 3-D multi- or single-user interactive and serious game creator. Individuals may “drag and drop” 3-D scenes from a gallery and create 3-D learning environments for distribution via the Web or on a Compact Disc Read-Only Memory (CD-ROM).The Carrick Foundation funds the ALIVE project and USQ’s Vice Chancellor’s Strategic Development Fund and continues to involve academics from a variety of disciplines [30]. De Byl [28], the team manager for the project, states the need for educators to dabble in 3-D virtual worlds and gaming applications as a method to promote interactive learning. The open, non-proprietary AliveX3D program is based on the Web 2.0 ethos of social networking and exemplifies the creation of a Web 2.0-Web3D hybrid platform which is “interoperable” and contains reusable 3D learning objects with an overall intention of “1) the re-use of data sources; 2) cost-effective scalability; 3) user controlled data; and 4) collective intelligence with respect to the e-Learning possibilities” (p. 6) [28]. Furthermore, when compared to the proprietary 3-D environments of SL and Active Worlds, AliveX3D, as an e-Learning application, has the capacity of being extended and allows educators the opportunity to control the content. It is relatively inexpensive to install and use because there are no licensing fees attached to the program [28].

Who would imagine attending medical school in a virtual world? For many years healthcare educators have developed online learning opportunities for medical and nursing students. Stott [33] reports universities are turning to SL’s virtual world and encouraging students to “fly” into 3-D lecture halls as “cybergoths.” Problem-based learning groups enrolled in a clinical management course at Coventry University meet in SL and are employed to build learning facilities for the next semester of SL students. This management course teaches students to manage healthcare facilities and is reported to be the first healthcare-related class to use SL as a learning environment. Another example of a medical school using SL is St. George’s Medical School in London. The technology enables students to interact with patient avatars in a simulated world and, moreover, students from around the globe may listen to invited guest lecturers in SL. The novel idea of combining medical simulation with gaming technologies is happening, and collaborators at Forterra Systems and the Summit group at Stanford University medical school are developing human avatars which exhibit life signs with the hope the learner’s active participation will encourage awareness, team cooperation, and decision-making skills [15].

Another virtual world project developed by staff at the Imperial College in London, in collaboration with the National Physical Lab in the United Kingdom, is the Second Health Project [34]. A detailed hospital comes to life in SL when physicians, writers, videographers, animators, designers, and builders gather together to create a fully equipped high technology system of healthcare that primarily focuses on health promotion while providing some detailed animations that simulate disease processes, such as heart attacks and other medical conditions. The community hospital is designed to represent real life in a modern UK city. Currently, the hospital is used for medical and other healthcare-related training. Mesko [35] presents the top 10 virtual medical sites in SL. For example, the Ann Myers Medical Center in SL is an environment where medical students may practice conducting physical exams and analyse radiological films, as well as learn how to detect heart defects. Boulos [36] developed The Sexual Health SIM in Second Life. Avatars may interact with different objects in an aesthetic “in-world” environment and learn about safe sex and sexual health topics (Figure 3).

The development and use of 3-D virtual worlds in nursing education is increasing. For example, students may learn how to provide step-by-step care for a patient suffering from chest pain via SL [1]. According to Miller [1], students build SL “objects” to show what they have learned. Furthermore, students collaborate with other students from different countries and other medical professionals. Miller uses “Centralia” island in SL as a place to teach nursing students (Figure 4).

**Figure 3:**
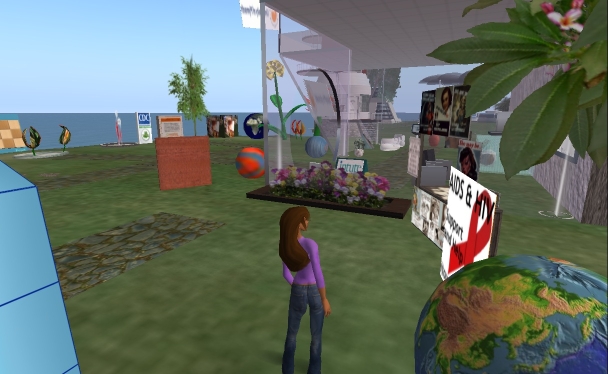
Maggie Waechter (the avatar of the author) visiting the Sexual Health SIM in Second Life [36]

**Figure 3:**
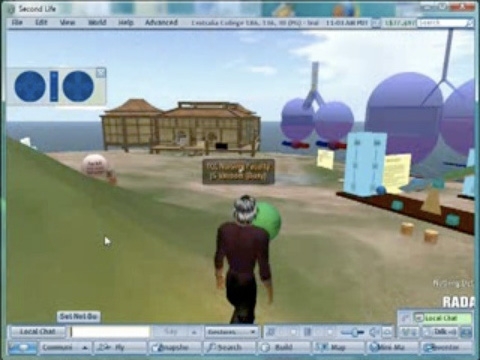
A screenshot of objects representing lungs in nursing education [37]

Objects representing lungs connected to large airway objects illustrate various lung disorders for student learning. Miller claims the 3-D objects, representing body parts, are easy to create. Learning objectives are provided for the students visiting “Centralia”, and critical-thinking questions are posed along with the anatomical objects. The student’s avatar may walk on different electrocardiogram (ECG) tracings on a 3-D floor and then names the represented rhythm. This is an example of immersive interactive learning. The site allows students to view objects in SL together and discuss different medical conditions in a team approach.

Another example of a cognitive, experiential 3-D learning tool is PULSE!! [38]. Researchers at PULSE!! state students respond positively to using the virtual world as a method to learn clinical skills and increase diagnostic thought processes. Of those polled, 80% of the participants stated the platform captured their interest, and 82% expressed positive thoughts about using PULSE!! The Office of Naval Research funds the program and Congress has appropriated more than US $12 million dollars to the project’s sustainability.

## Practical and Useful Theories Expressed for the Use of 3-D Learning Environments

### Roger's Diffusion of Innovations Theory

When an academic or learner is introduced to a specific innovation which may influence their intellectual prowess, an assumption may be made that the innovation has inherent characteristics, such as the creation of opportunities for enhanced learning, versatility, simplicity, and enhanced, self-regulated learning. Many academics are time poor, and the thought of mastering a new e-learning tool, purported to enhance student learning, may create more stress. Barriers begin to develop between the educator and the new learning tool because there is a lack of time to learn and understand how to use the new tool. Often there is very little evidence of positive outcomes when an innovation is newly introduced. The advantages of the new technology may be introduced; however, the “why,” “what,” and “what about” questions regarding the innovation need to be answered in order for the technology to be accepted, experimented, and adopted. Rogers [5] explains five attributes having an effect on an individual’s decision to adopt an innovation. These include a) the relative advantage of the innovation over an idea that it supersedes; b) compatibility, or how does the innovation meet the needs of potential adopters; c) complexity, or how difficult the innovation is to understand and use; d) trialability, or how the innovation may be tested in a timely fashion; and e) observability, or, in other words, the outcomes associated with the innovation are visible to others.

Grant foundations and university development funds have supported the development costs associated with educational virtual worlds [39]. Wright states the development costs for a SL virtual campus is approximately US $25,000 if constructed by a commercial agency [39]. Even though there is an increased demand for gaming, animation, and 3-D visual spaces in higher education, there needs to be clear explanations to students, academics, and employers, about the benefits associated with virtual worlds. Professional skills, such as reasoning, teamwork, and role playing, will need to be transferred to the workplace before adoption is easily accepted [17,29].

Historically, 3-D learning-environment development required complex and highly involved elements of software engineering and computer science [40]. For many educators, an online 3-D learning environment would not be perceived as being an advantage because the “plasticity” associated with the virtual worlds—or, in other words, the ease of constructing and changing the learning environment as necessary would not be understood until the educator played and experimented with the virtual worlds [27]. The valuable communication tools built into the 3-D system, and the ease associated with the learning area to provide guidance and assistance, may not be fully realized. In addition, some educators still do not see e-learning beyond learning management systems and have not had exposure to virtual worlds (eg, played in them). Therefore, a low compatibility issue presents itself in the academic milieu. Rogers [5] explains three types of decisions associated with adopting an innovation: a) the system is “optional” and the individual may adopt or reject the technology being considered, b) the collective group makes a decision to adopt or reject the technology, or c) one individual in authority makes the decision with the idea the group will follow that decision. These types of decisions have a large impact on the individual adopter and the outcome for the social system as a whole. However, the tides are currently changing with Google Corporation’s release of “Lively” [40], in which individuals can easily create their own avatar and personal room to embed at a location of choice on the Web (eg, blog, social network site, or Web page). Google’s goal is to create a massive virtual world where Google account holders’ avatars may visit at any time and interact with one another. Perhaps educators may use this new social network tool to create class “rooms” and take advantage of the ability to embed YouTube videos on virtual plasma screens. Perhaps the idea of creating a 3-D learning environment may no longer be perceived as a daunting task due to Web 2.0 advanced technologies.

### Siemens Connectivism: A Learning Theory for the Digital Age

Siemens’ [6] Connectivism Learning Theory is about the formation of “connections” and how, from these connections, a building of “networked” learning occurs. Individuals are continuously forming social networks and are being acted upon, or act upon, while moving in and out of these networks. “Living life” is a dynamic learning process, and we are constantly developing new connections, moving toward larger networks or breaking down into smaller groups, as we interact with one another. Furthermore, the means of adapting and learning while interacting with the world around us are ever changing. Learning theories attempt to explain the complexity of learning, and many educators are moving away from the static knowledge development or the “destination” of knowledge and are embracing a paradigm shift that is one of “a process of walking in varying degrees of alignment with a dynamic environment” (p. 1) [6]. Therefore, connectivism dovetails nicely with the thesis of virtual 3-D learning environments being supportive of communication, community, and sharing. Communication is an aspect of effective teamwork, while another important aspect of teamwork that is essential in healthcare and education is “how” members interact with each other or develop connections that form a community of practice. Hobbs, Brown, and Gordon [40] state the benefits associated with developing communities of practice within virtual world environments in order to transfer skills that will enhance collaborative work within the work environment. Moreover, there exists a commonly held belief students will feel more satisfied with their course work if they are involved and continue to develop relationships with their peers in learning environments. Virtual world environments allow for the transfer of skills from virtual worlds to the work place and perhaps the development of lifelong skills.

## Strengths Associated with 3-D Virtual Worlds

Virtual worlds have been portrayed in film and literature for many years and may play a role in education, business, and healthcare education because this technology may change the way people learn and live in the future [13,17,29]. The major strengths associated with virtual worlds are one’s ability to design and construct unique environments and then share them with others in a collaborative fashion. Educators may write specific learning goals for students to complete while learners actively build and interact in environments that promote creativity and social networking. Sibbet [21] outlines how virtual worlds are “reshaping” learning, communications, social interactions, and perceptions. Furthermore, Sibbet presents interesting questions surrounding themes, such as cross-generational communications, identity exploration, cross-cultural exchange, problem solving, deep dialogue, and ceremony. All of these questions have implications for healthcare professional development and education, and even healthcare delivery.

Since an online virtual world is available 24/7 there is an anytime/anywhere benefit for distance education students. There are other advantages, such as virtual training approaches that yield results and are invaluable for healthcare professionals, and, for the healthcare consumer, there is an advantage of logging on and learning from events happening in SL, such as the 2006 health fair. This is especially useful if the individual is at a physical distance. The medium is excellent for improving students’ access to places otherwise difficult to reach. The technology makes spatial representation useful for hands-on learning and heightened student engagement because the real-time social interaction and gaming aspect spurs chances for “discovery-based and goal-oriented learning” [29].

## Challenges Associated with 3-D Virtual Worlds

With any innovative technologically supported pedagogical tool there are critics who debate the usefulness of the application [4]. However, one recalls when critics questioned the validity and reliability of the stethoscope invented by Laennec in 1816 and how today it is second nature to use this assessment tool. Hence, using virtual simulations to teach healthcare students may be questionable until more research is conducted and educational outcomes are realized. Two of the major challenges associated with virtual worlds in education is the time involved in creating learning spaces within a virtual environment, as well as the cost involved. Blake argues since virtual world games are played in real time, the flexibility of distance-learning tools is not realized. The efficiency associated with sharing text, images, and videos via an avatar versus a standard format on a computer’s desktop is questionable (Adam Blake, written communication, October 19, 2007). A negative human response to other avatars in the learning environment is possible; however, this may exist in traditional learning settings as well. The allure of the dynamic colourful SL environment may distract the student’s attention from the learning objectives. Of course, these are all points of view that may be debated. Clearly empirical research is needed for future use of virtual worlds in healthcare training and general education. Striking challenges facing developers of virtual worlds and serious gaming for educational purposes are ownership of collaborative work and certification of authorship. Both of these issues pose a problem for evaluation for learning outcomes [29].

## Research Efforts

Educational research regarding 3-D virtual worlds and the effects on learning outcomes is lacking. However, Bainbridge [13] states scientists and scholars are moving forward in conducting research about virtual worlds and encourages this research be completed in a timely manner because the current transformation of the virtual worlds is time sensitive, and future retrieval may be challenging. Ackermann, as stated in Schneider [41], explains interactivity is a key to learning. “An increasing number of software designers, cognitive scientists, and educators have come to the view that experience is actively constructed and reconstructed through direct interaction with the world, and that, indeed, knowledge is experience” (p. 1) [41]. Byrne [42] conducted a pre- and post-experimental study examining the use of Virtual Reality (VR) as an educational tool and discovered high-school students’ (N=38) knowledge of atomic and molecular structures before and after a VR experience was significant for “interactivity” but not for “physical immersion.” Statistical results for the aforementioned study were not presented. Chapman, Stone, and Nelson [43] indicate that providing simulations in 3-D virtual learning environments presents the potential of enhanced learning. Increased interactivity with the learning material, provision of self-directed and immersive learning experiences, and students’ co-creation of learning content with other students and the instructor are cited in the literature [43]. The same researchers [43] explain simulations and case-based scenarios build upon well-defined educational theories, such as constructivism, experiential learning, adult learning theory, social presence, and situated learning. The considerations determined by researchers are: a) resources to develop the virtual worlds, b) available technical support, c) ethical considerations, d) accessibility, e) usability concerns, f) ownership of content, and g) peer review. More research is needed to determine students’ satisfaction, competency, and knowledge acquisition.

## Vision for Future Use in Medicine and Healthcare Professional Education

One may view online virtual worlds and serious gaming as a threat to the adoption and purchase of high-fidelity computerized patient-simulation mannequins that are currently purchased for healthcare-profession training. For example, nurses may login into SL and learn Advanced Cardiac Life Support at their convenience, and it costs virtually nothing for the nurse and perhaps a nominal fee for the developer. Why would an educator want to recreate or even offer such a training opportunity when it already exists on the Internet? Of course, one could argue that it lacks a haptic quality essential for the procedure or lacks one-on-one assessment of skill acquisition. The educational opportunity in SL may not be a replacement for the doctor- or nurse-patient interaction or relationship, but SL may serve as an adjunct or pre- or post-learning tool. The advisory board responsible for Educause’s Horizon Report [29] states collaborative learning experiences taking place in virtual worlds today are easier to find than a year ago when the authors predicted virtual worlds being one to two years away from adoption.

## Discussion

Critical challenges associated with the development, adoption, and evaluation of online virtual worlds for healthcare training education do exist. However, there is an underlying push in higher education to adopt these collaborative tools and shift the paradigm from a traditional Socratic method of education to one possessing a more active and interactive nature [29]. Virtual worlds are a part of our present existence and offer online users of all ages opportunities to explore, create, imagine, collaborate, role play, interact, socialize, learn, and experience moments in a safe and vivid manner. What better way to learn than when you are having fun and actively participating in making choices, decisions, and interacting with others in a safe space? Let’s look forward and determine research methodologies, such as experimental or qualitative design, to evaluate the relevance of virtual worlds for teaching, learning, and creative expression. Research will substantiate factors we suspect have an influence on a learner’s ability to retain and search for and retain knowledge. The educator is in a position to look at who the learners are and what the learners really want from their learning experiences. What motivates generations Y and Z to learn? What are they accustomed to doing on a daily basis? Why not research, investigate, and try this social networking and virtual reality tool and create learning moments in what may really be a “now” in-world?

## Conclusion

Virtual 3-D learning environments may encourage active learning while students create and explore activities similar to those of a “field trip”, versus the experience of a static classroom setting. This reaching out and meeting new avatars and practicing communication skills in an aesthetic environment may help maintain today’s students’ interest in learning and provide valuable experiences that may enhance student engagement, promote participation, and motivate self-directed learning. Educators that see “on-the-horizon technologies” in higher education present an opportunity for today’s learners to explore exciting worlds beyond the traditional classroom and are showing an understanding of current students’ use of technology. Moreover, participating or playing in a virtual world may be enjoyable for the learner, encourages creative expression, broadens socialization skills, promotes independent problem solving, provides opportunities for self-teaching, and sets the stage for group work. There are established opportunities for educators to network with alliances that are already developing, implementing, and researching 3-D virtual worlds as learning spaces [1,21,27,28,38]. Therefore, the wheel of technology does not need to be reinvented by individual educators because, as outlined in this paper, there are opportunities for educators to meet like-minded individuals. Empirical research findings will help determine if learning objectives are met by offering this type of educational tool. Some educators may balk at adopting this technology because there is a learning curve associated with the use of 3-D virtual worlds. It is noteworthy to understand the necessity to educate the educator in order to bring that willing individual up to speed on how to operate the 3-D virtual world. However, there are some very current Web 3-D programs, such as Lively [40], that may decrease the learning curve and motivate the educator to develop class “room” spaces. Let’s have fun, explore these fascinating worlds and games, and network with others while respecting diverse ways of life-long learning and current researchers’ findings.

## Multimedia Appendix

From Computer Based Simulations to 3-D Virtual Learning: Building Bridges to Collaborative Learning Spaces

## Abbreviations

3-D: three-dimensional
ALIVE: Advanced Learning and Immersive Virtual Environment
MMOG: Massively Multiplayer Online Game
MMVW: Massively Multiplayer Virtual World
SL: Second Life
