# Supplementary material for: Versatile, Immersive, Creative and Dynamic Virtual 3-D Healthcare Learning Environments: A Review of the Literature
Source: J Med Internet Res. 2008 Sep 1;10(3):e26. doi: 10.2196/jmir.1051 (PMC2626432; doi:10.2196/jmir.1051)
Supplement: Supplementary file 1 [file jmir_v10i3e26_app1.ppt]

## Slide 1
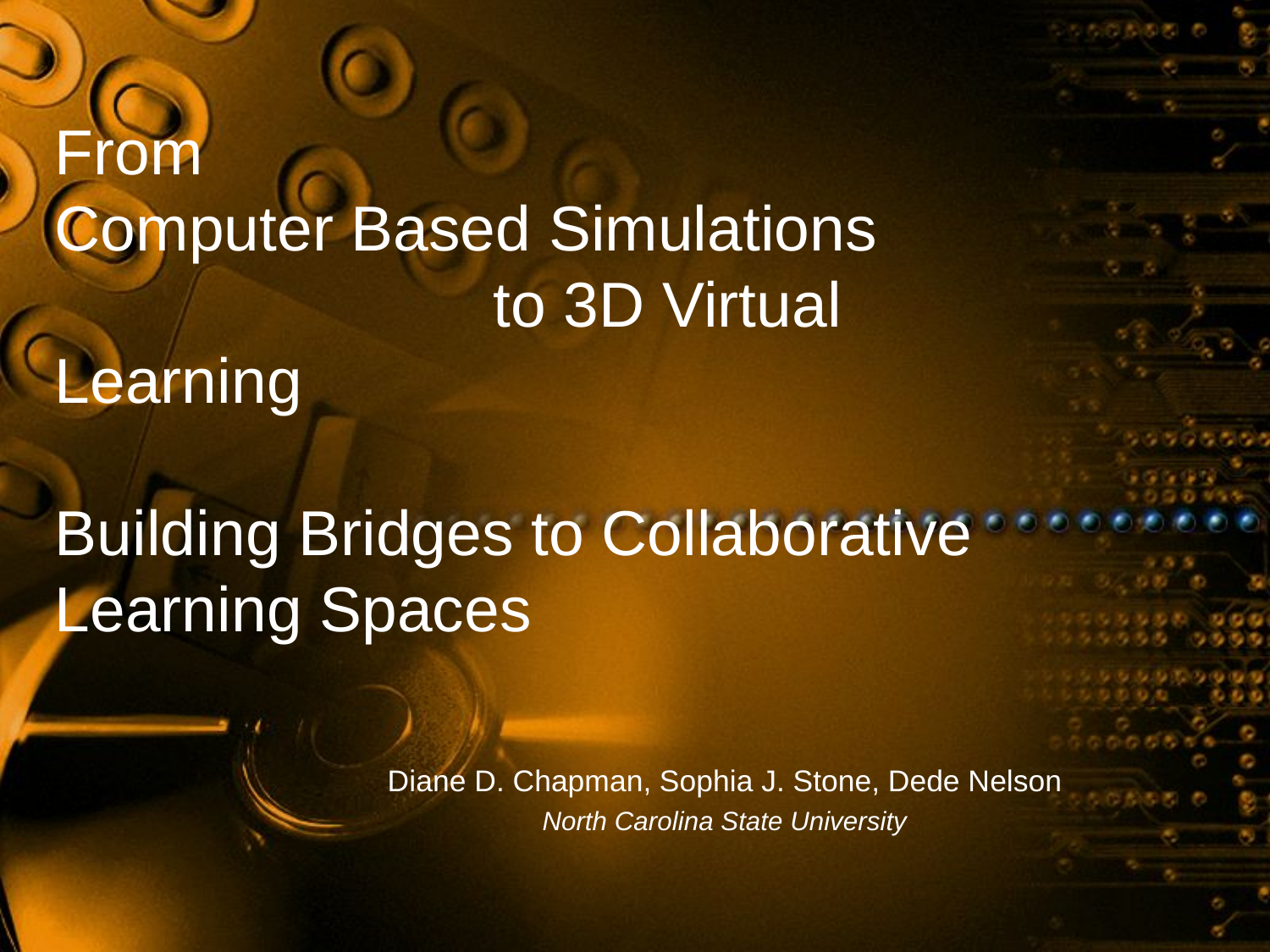

# From Computer Based Simulations to 3D Virtual Learning Building Bridges to Collaborative Learning Spaces
Diane D. Chapman, Sophia J. Stone, Dede Nelson
North Carolina State University

## Slide 2
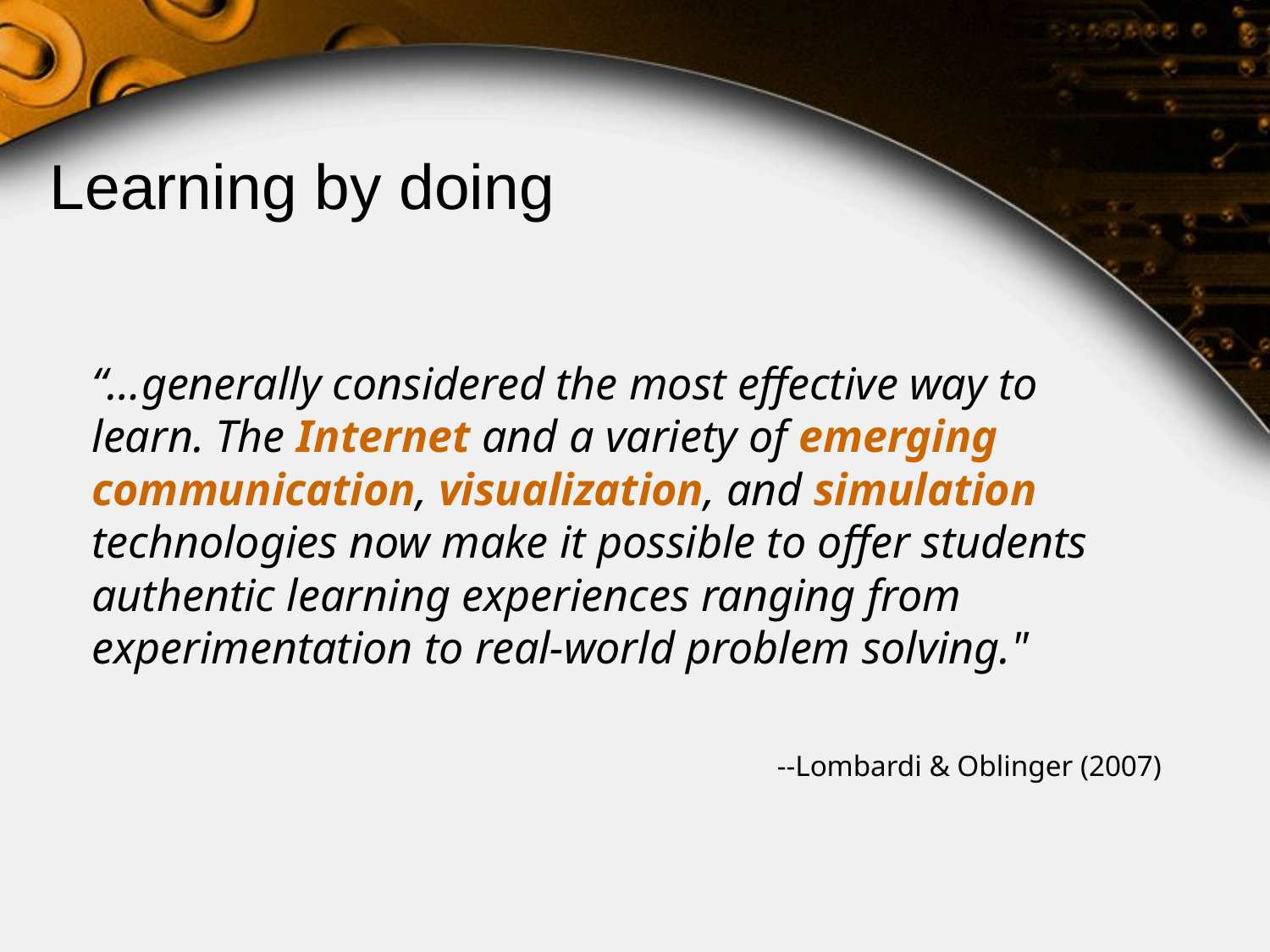

# Learning by doing
“…generally considered the most effective way to learn. The Internet and a variety of emerging communication, visualization, and simulation technologies now make it possible to offer students authentic learning experiences ranging from experimentation to real-world problem solving."
 --Lombardi & Oblinger (2007)

## Slide 3
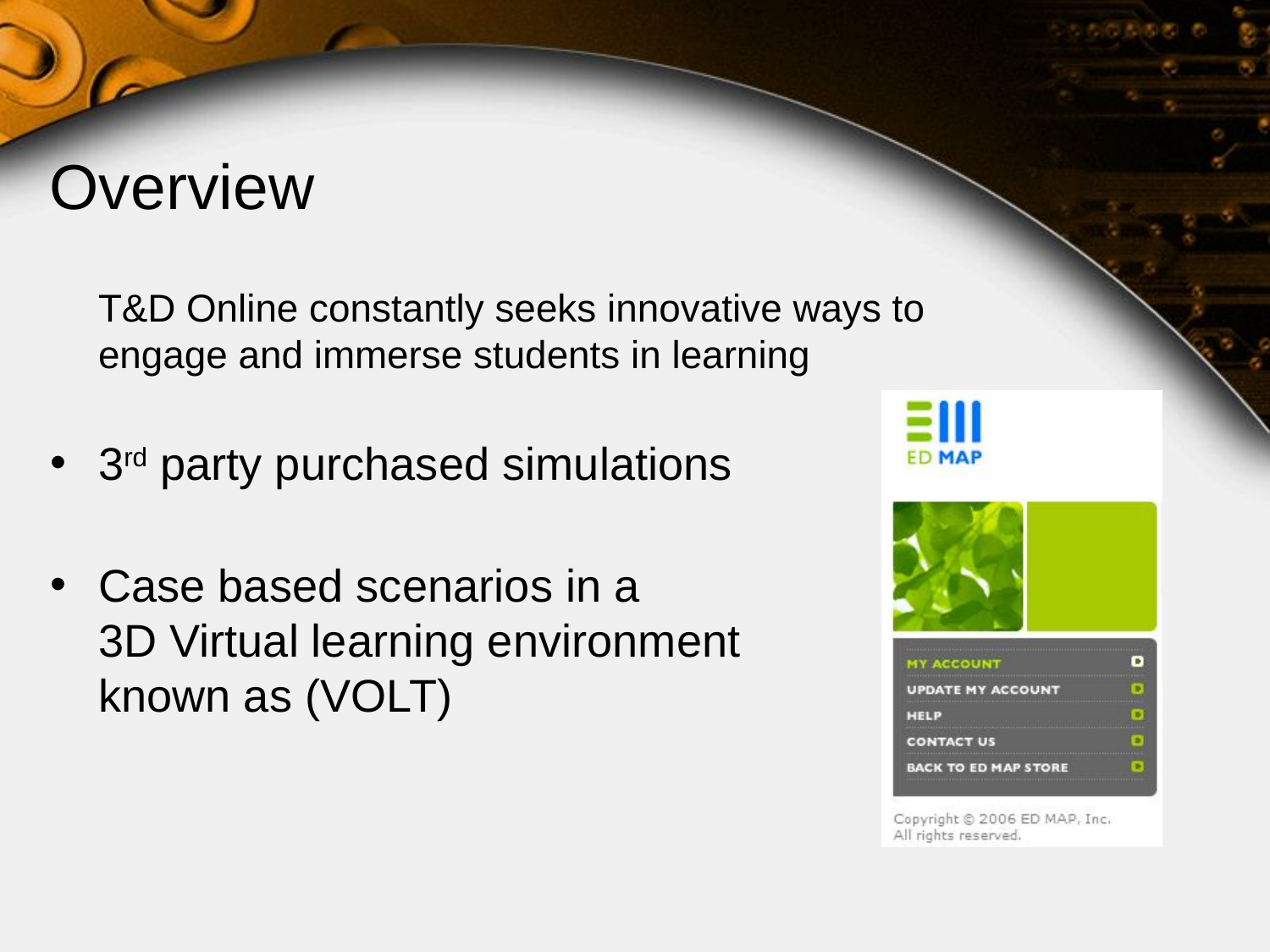

# Overview
T&D Online constantly seeks innovative ways to engage and immerse students in learning
3rd party purchased simulations
Case based scenarios in a
3D Virtual learning environment
known as (VOLT)

## Slide 4
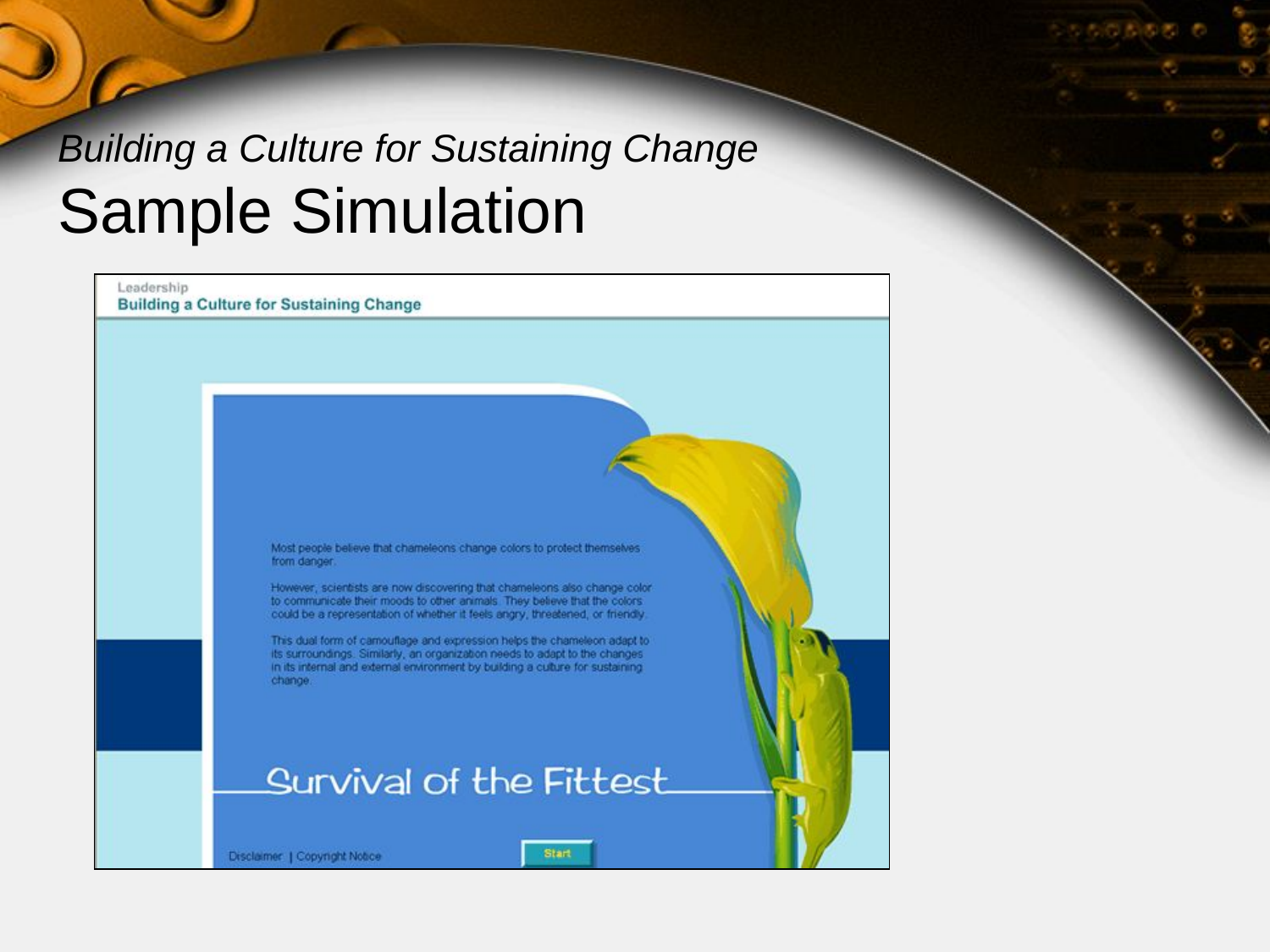

# Building a Culture for Sustaining Change Sample Simulation

## Slide 5
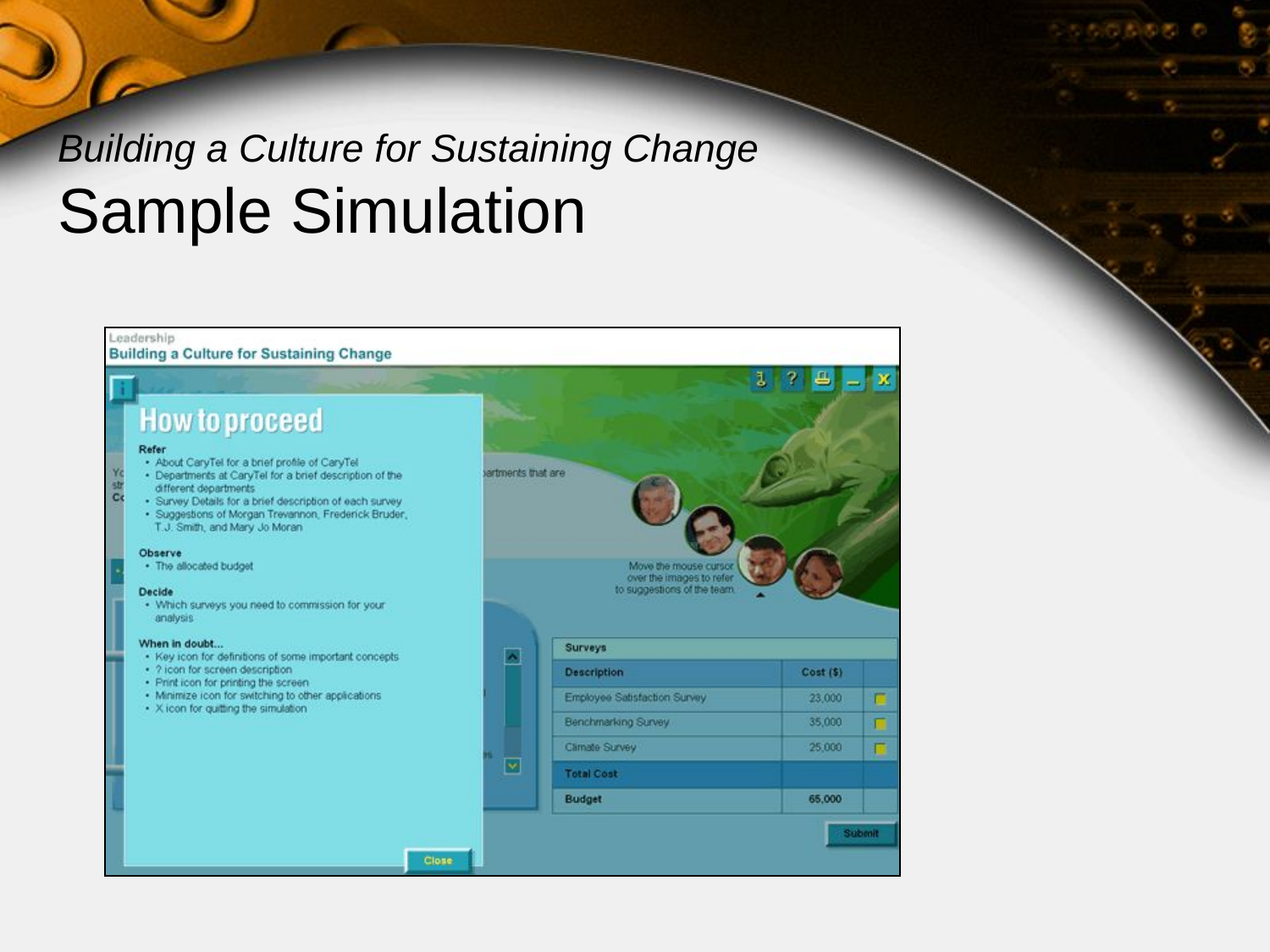

# Building a Culture for Sustaining Change Sample Simulation

## Slide 6
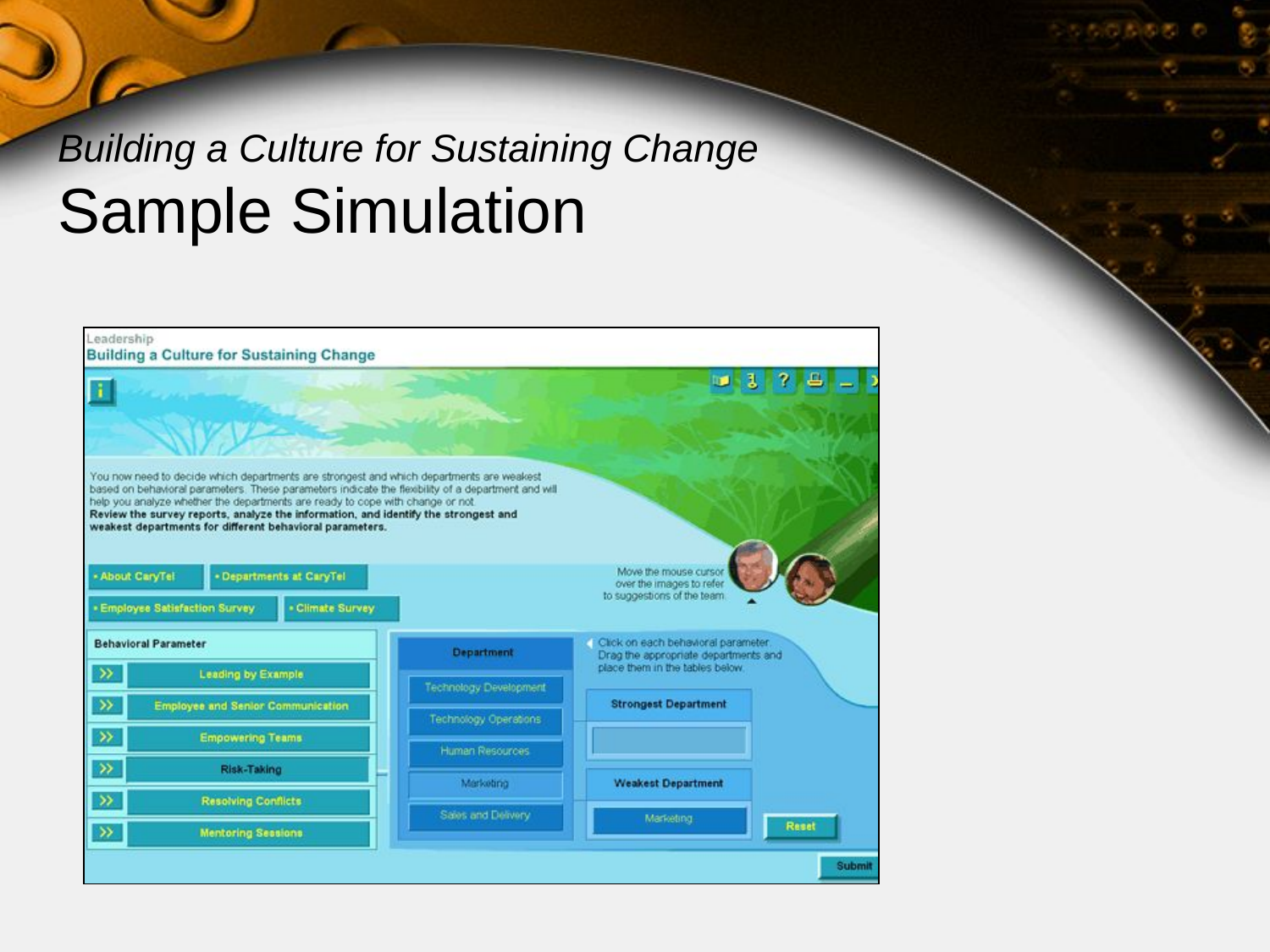

# Building a Culture for Sustaining Change Sample Simulation

## Slide 7
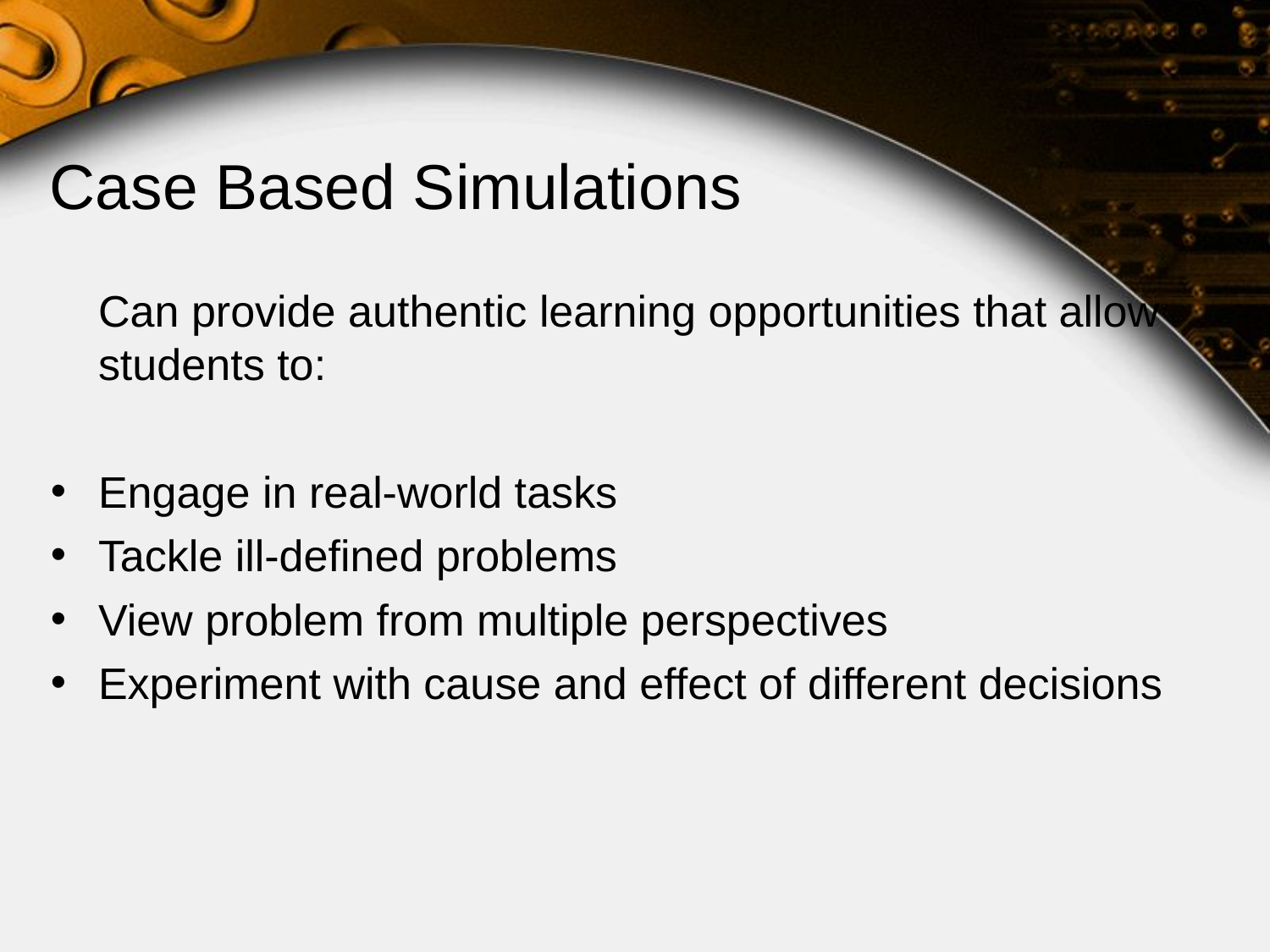

# Case Based Simulations
Can provide authentic learning opportunities that allow students to:
Engage in real-world tasks
Tackle ill-defined problems
View problem from multiple perspectives
Experiment with cause and effect of different decisions

## Slide 8
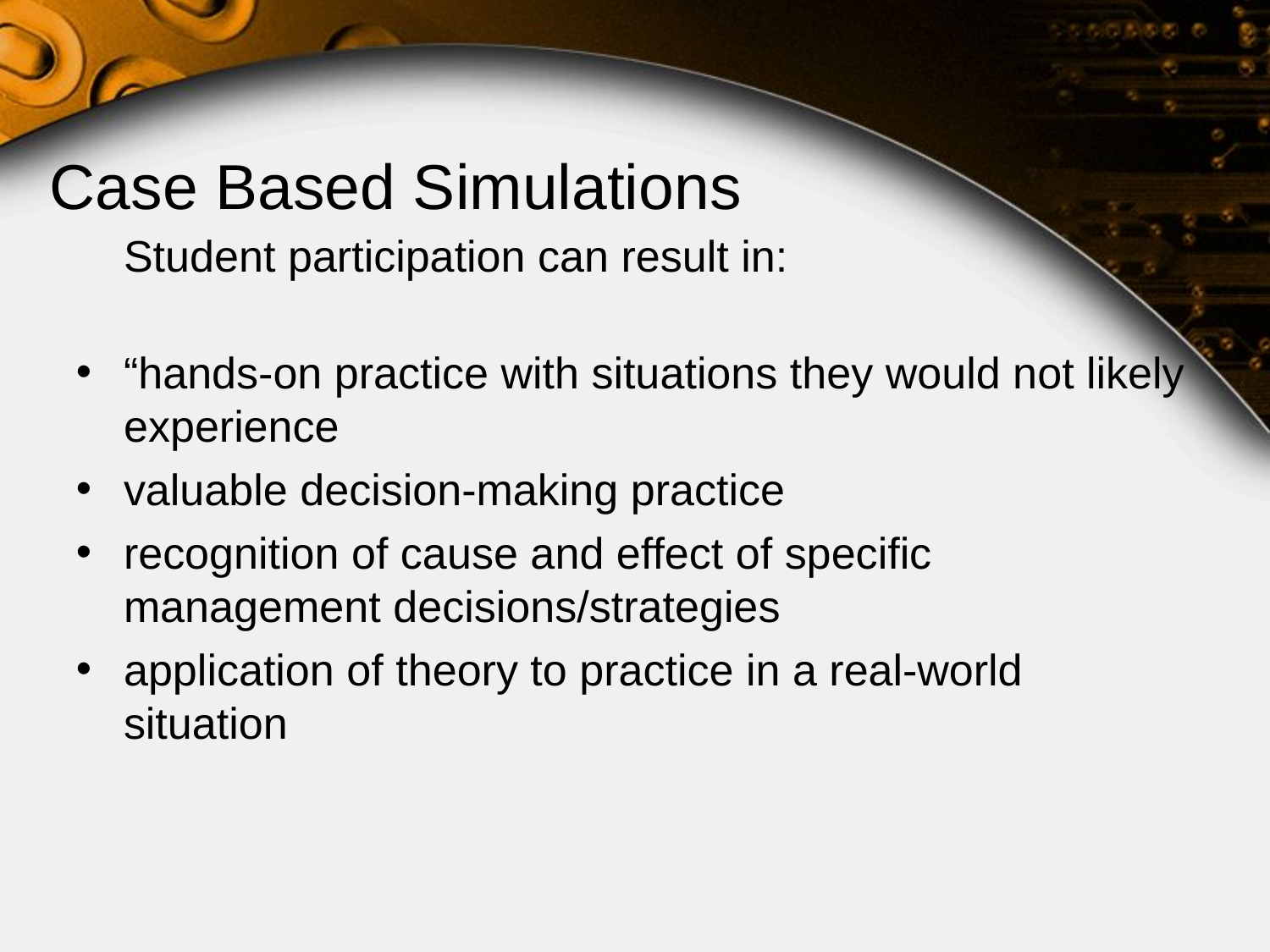

# Case Based Simulations
Student participation can result in:
“hands-on practice with situations they would not likely experience
valuable decision-making practice
recognition of cause and effect of specific management decisions/strategies
application of theory to practice in a real-world situation

## Slide 9
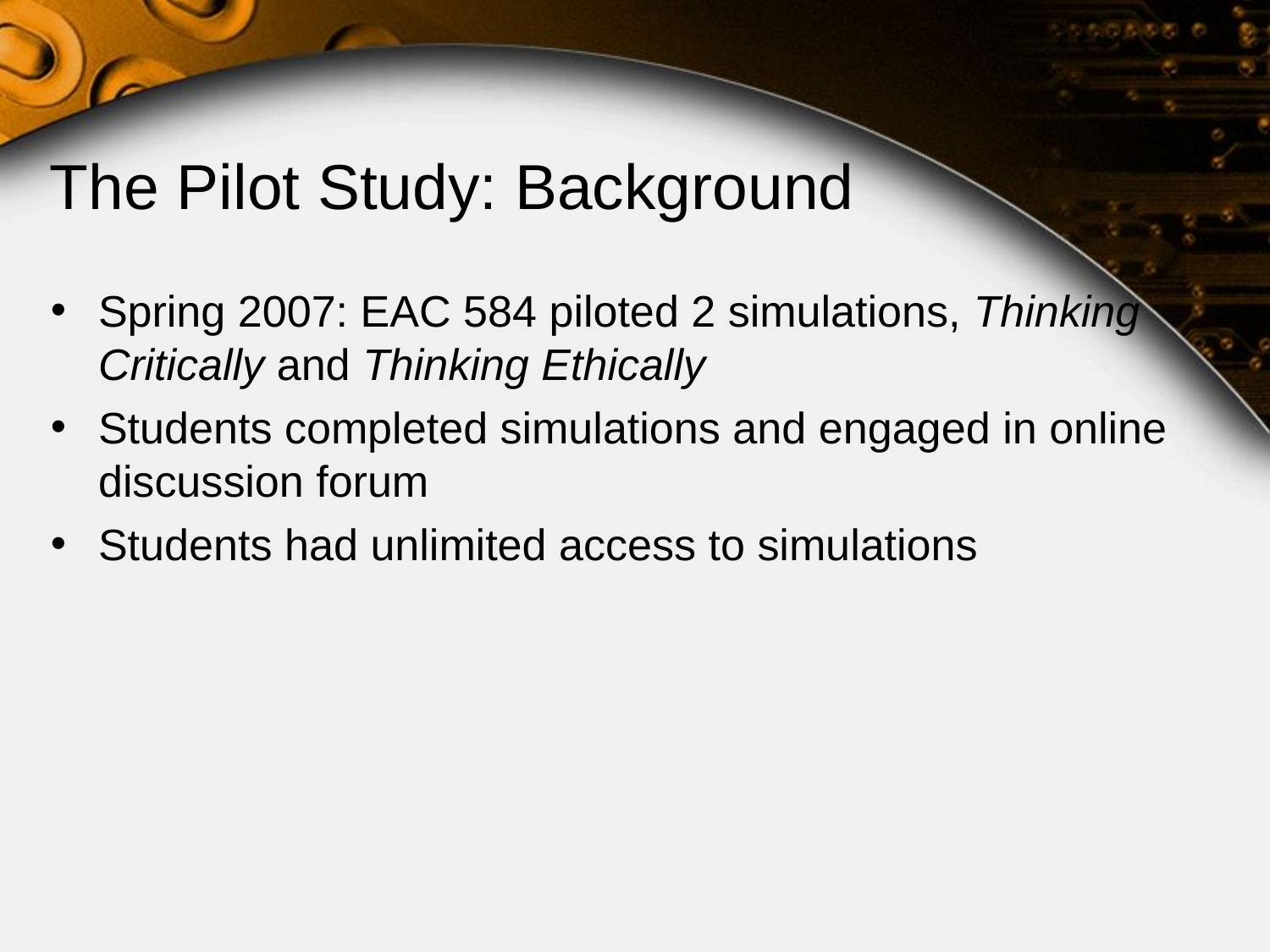

# The Pilot Study: Background
Spring 2007: EAC 584 piloted 2 simulations, Thinking Critically and Thinking Ethically
Students completed simulations and engaged in online discussion forum
Students had unlimited access to simulations

## Slide 10
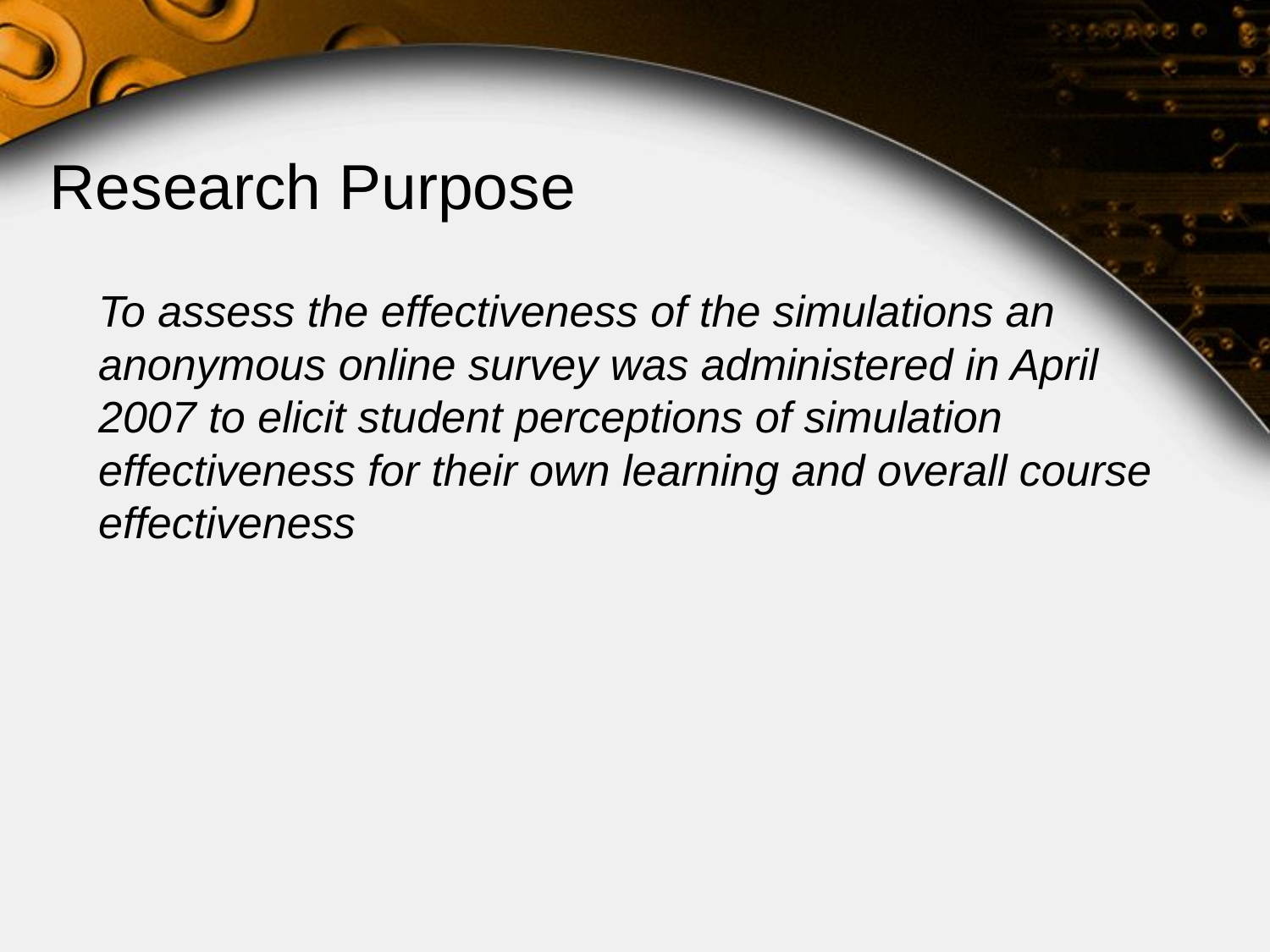

# Research Purpose
To assess the effectiveness of the simulations an anonymous online survey was administered in April 2007 to elicit student perceptions of simulation effectiveness for their own learning and overall course effectiveness

## Slide 11
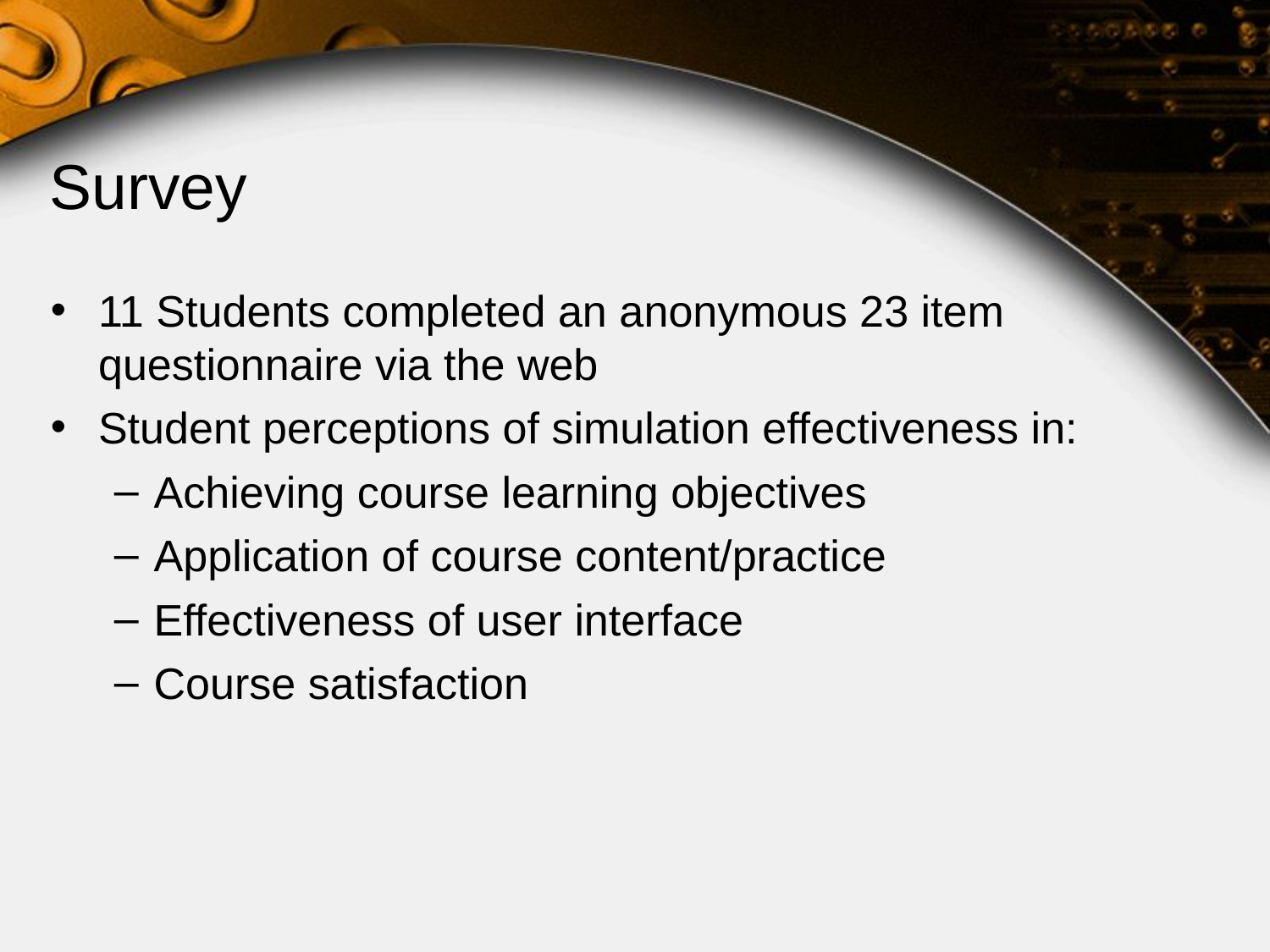

# Survey
11 Students completed an anonymous 23 item questionnaire via the web
Student perceptions of simulation effectiveness in:
Achieving course learning objectives
Application of course content/practice
Effectiveness of user interface
Course satisfaction

## Slide 12
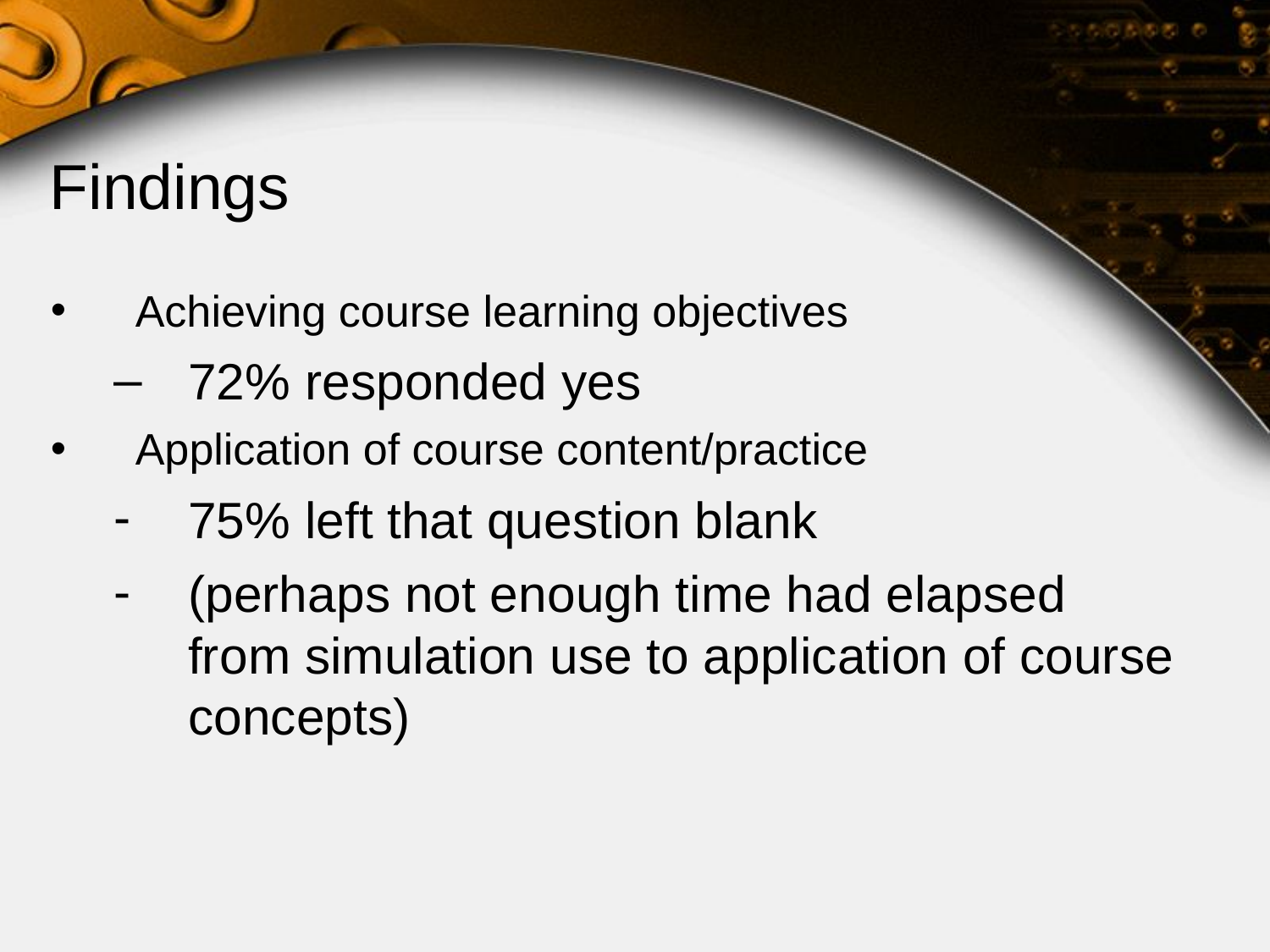

# Findings
Achieving course learning objectives
72% responded yes
Application of course content/practice
75% left that question blank
(perhaps not enough time had elapsed from simulation use to application of course concepts)

## Slide 13
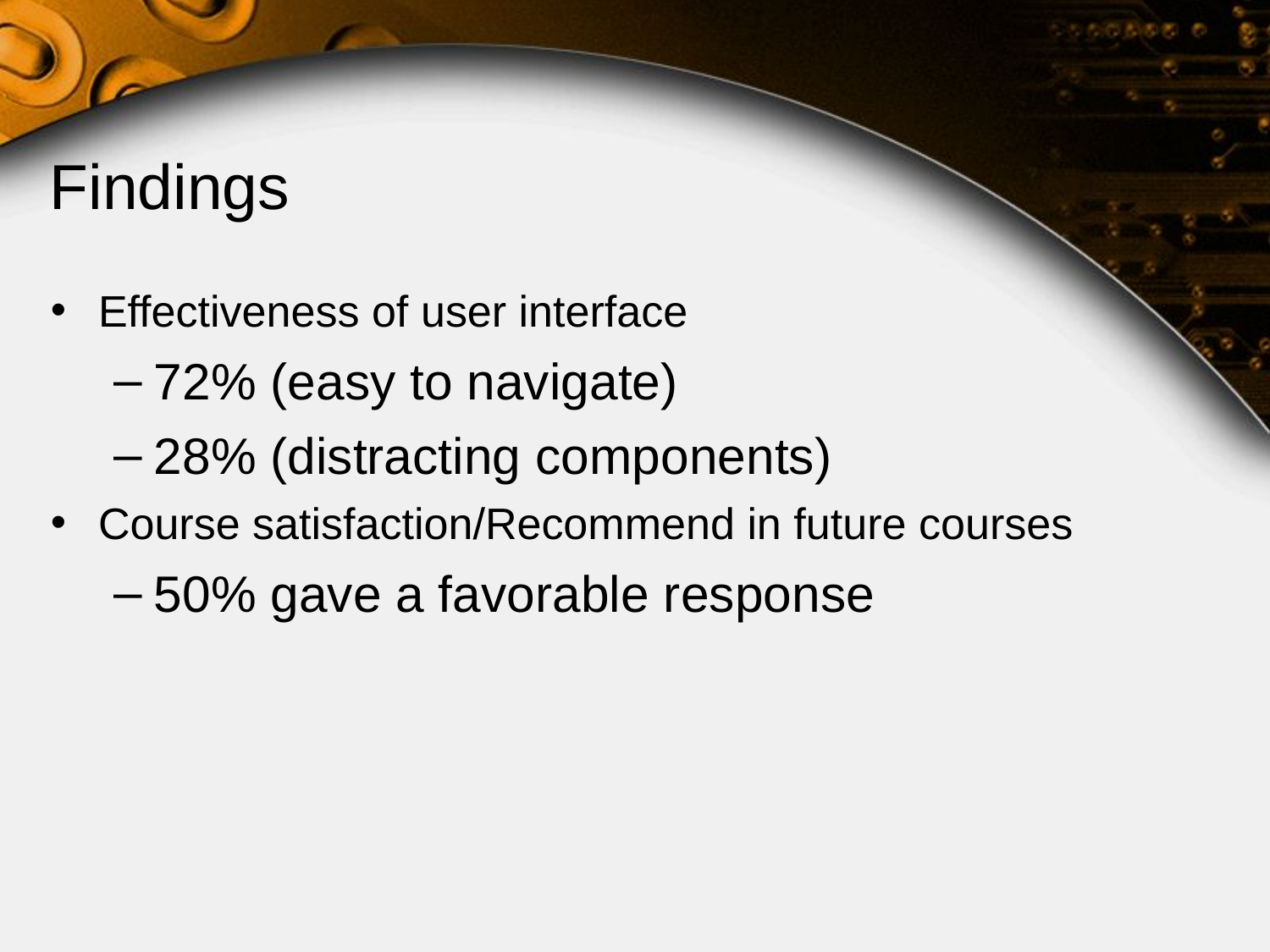

# Findings
Effectiveness of user interface
72% (easy to navigate)
28% (distracting components)
Course satisfaction/Recommend in future courses
50% gave a favorable response

## Slide 14
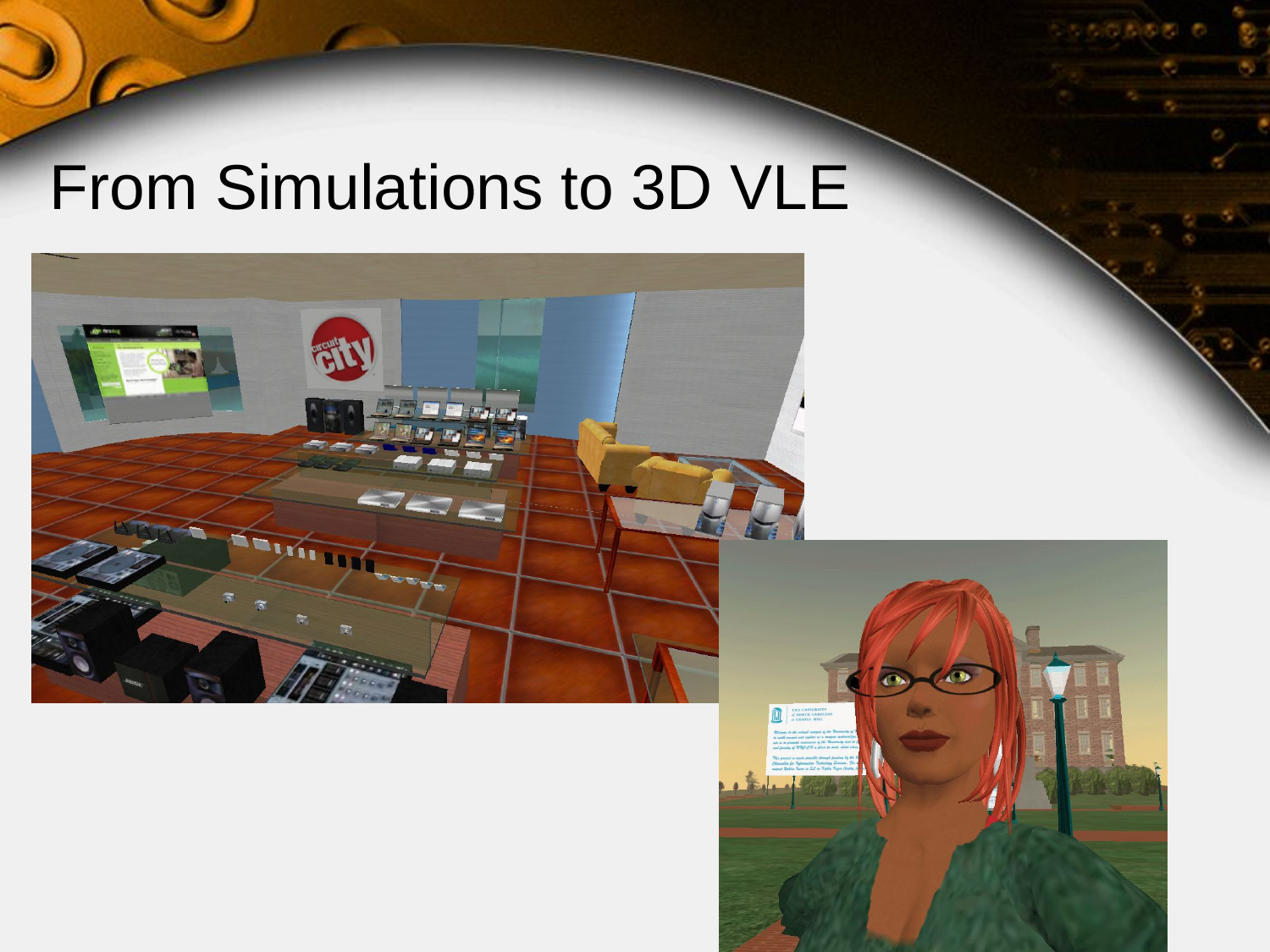

# From Simulations to 3D VLE

## Slide 15
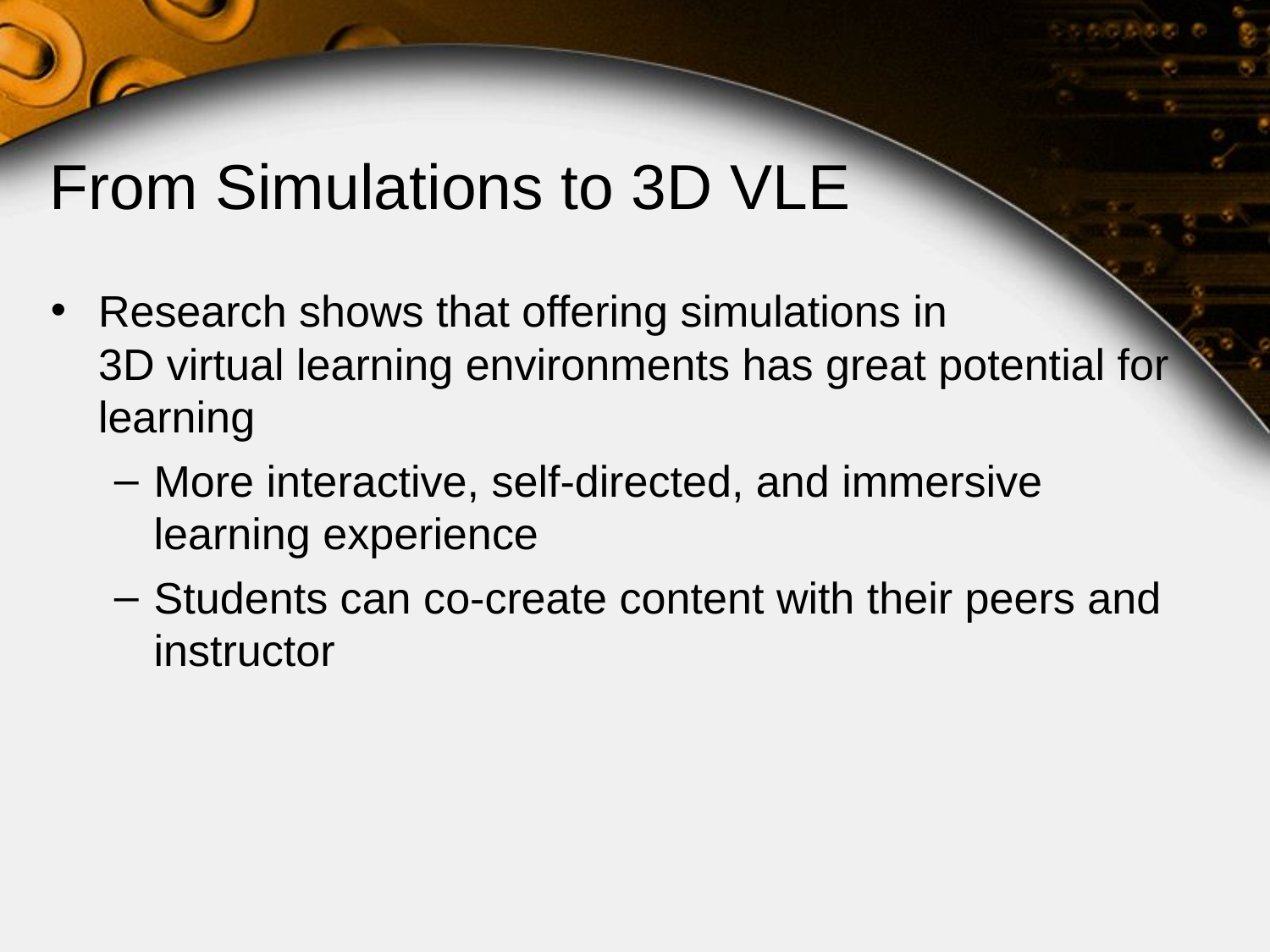

# From Simulations to 3D VLE
Research shows that offering simulations in 3D virtual learning environments has great potential for learning
More interactive, self-directed, and immersive learning experience
Students can co-create content with their peers and instructor

## Slide 16
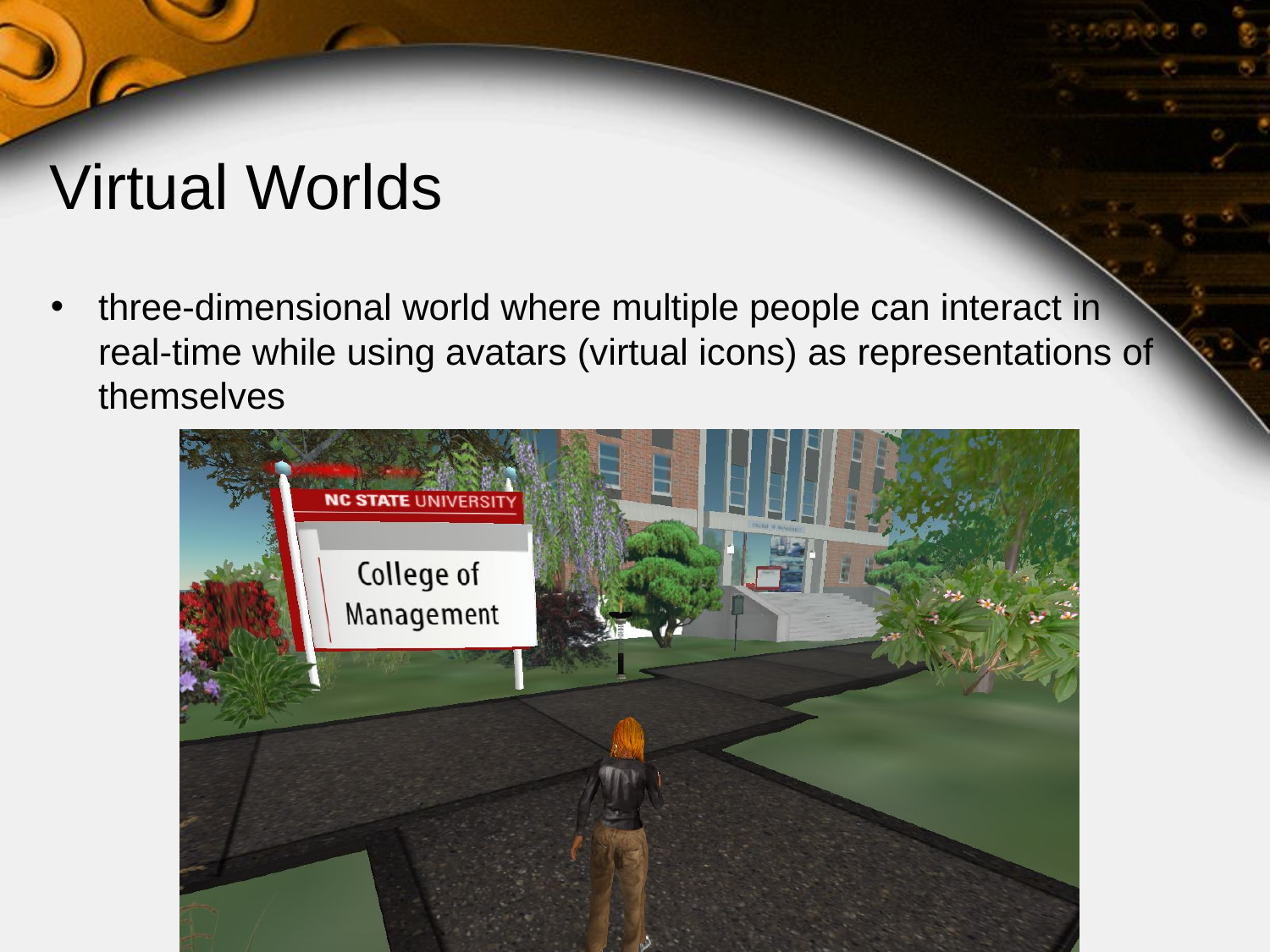

# Virtual Worlds
three-dimensional world where multiple people can interact in real-time while using avatars (virtual icons) as representations of themselves

## Slide 17
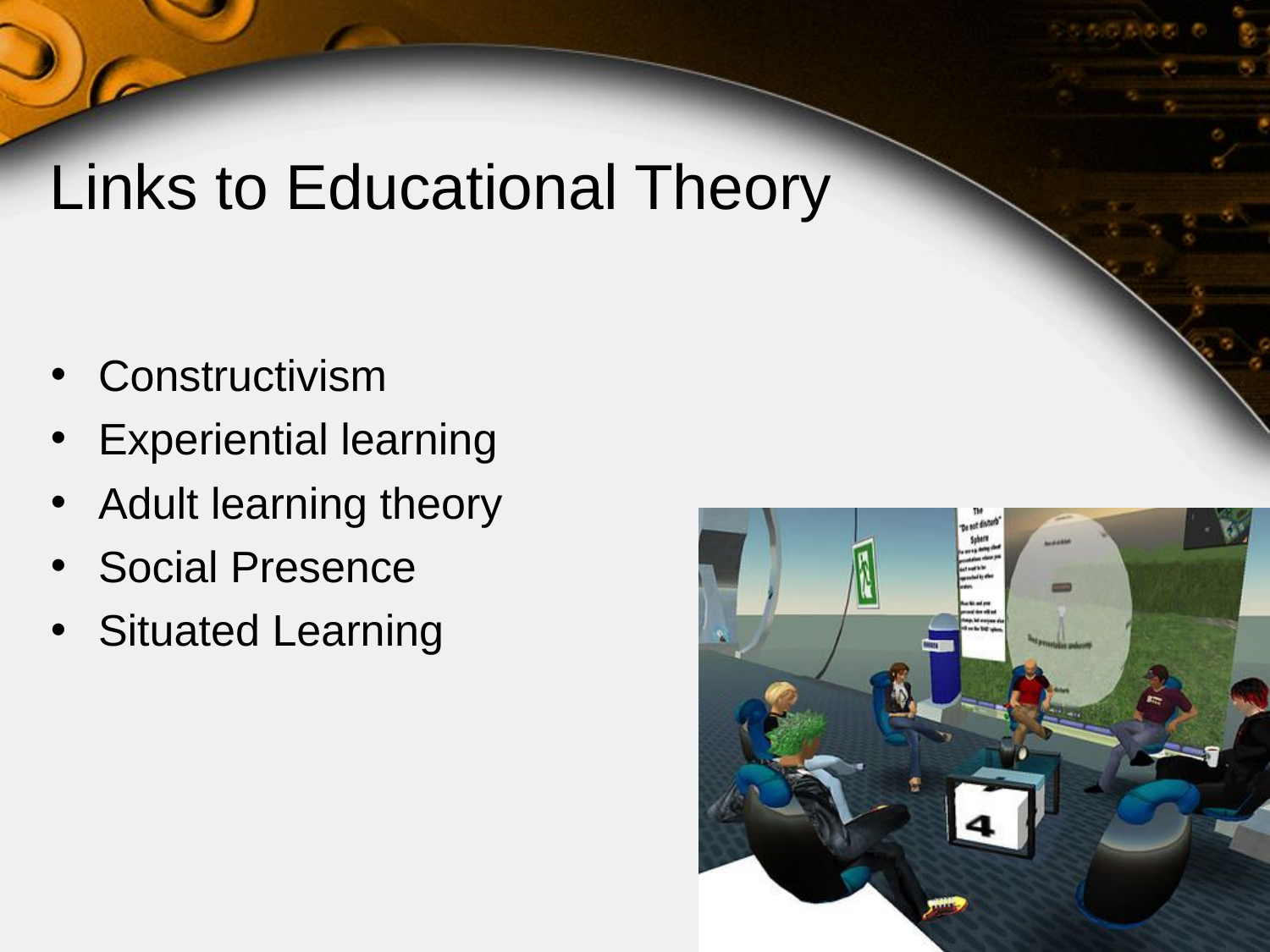

# Links to Educational Theory
Constructivism
Experiential learning
Adult learning theory
Social Presence
Situated Learning

## Slide 18
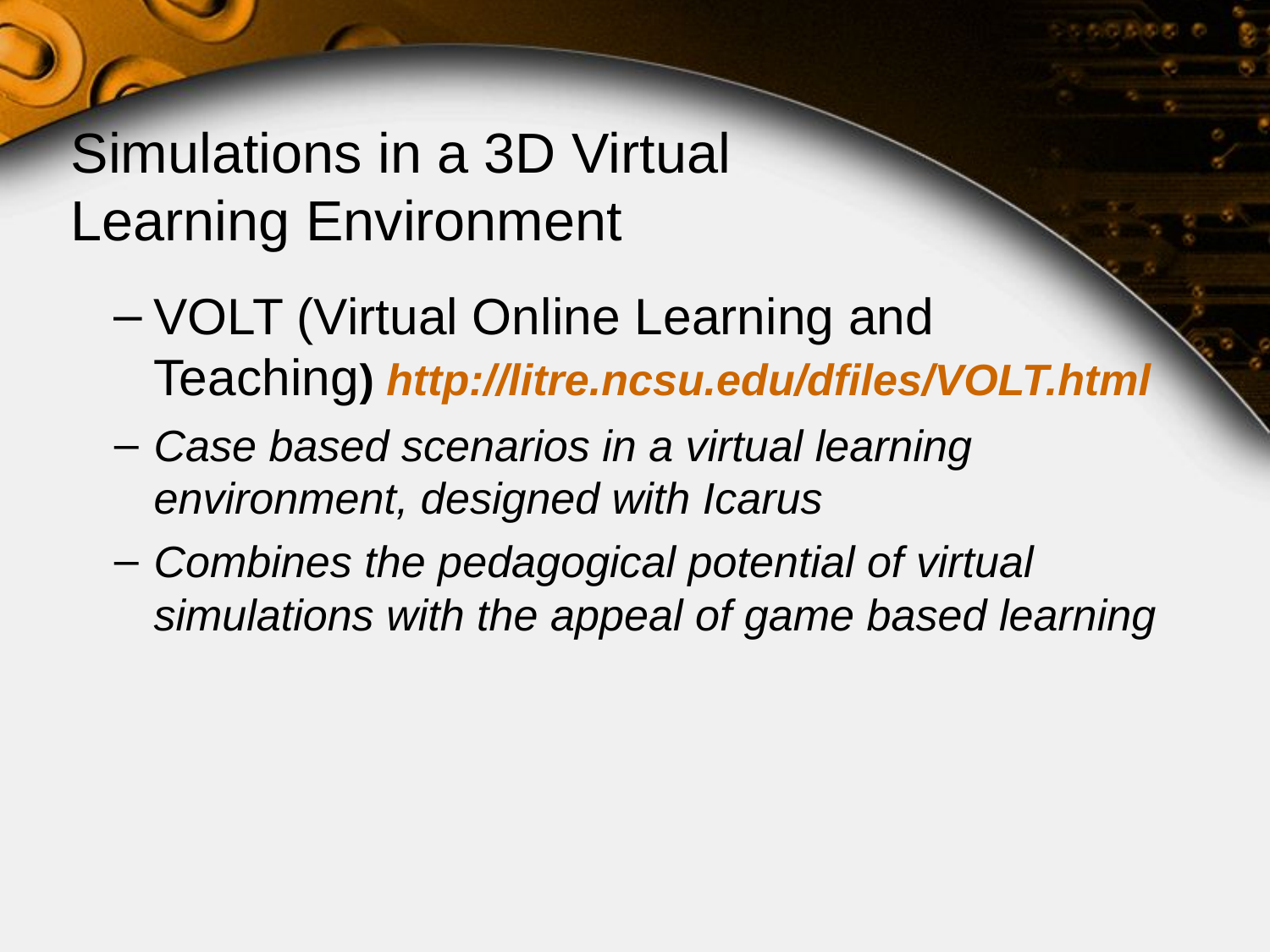

# Simulations in a 3D Virtual Learning Environment
VOLT (Virtual Online Learning and Teaching) http://litre.ncsu.edu/dfiles/VOLT.html
Case based scenarios in a virtual learning environment, designed with Icarus
Combines the pedagogical potential of virtual simulations with the appeal of game based learning

## Slide 19
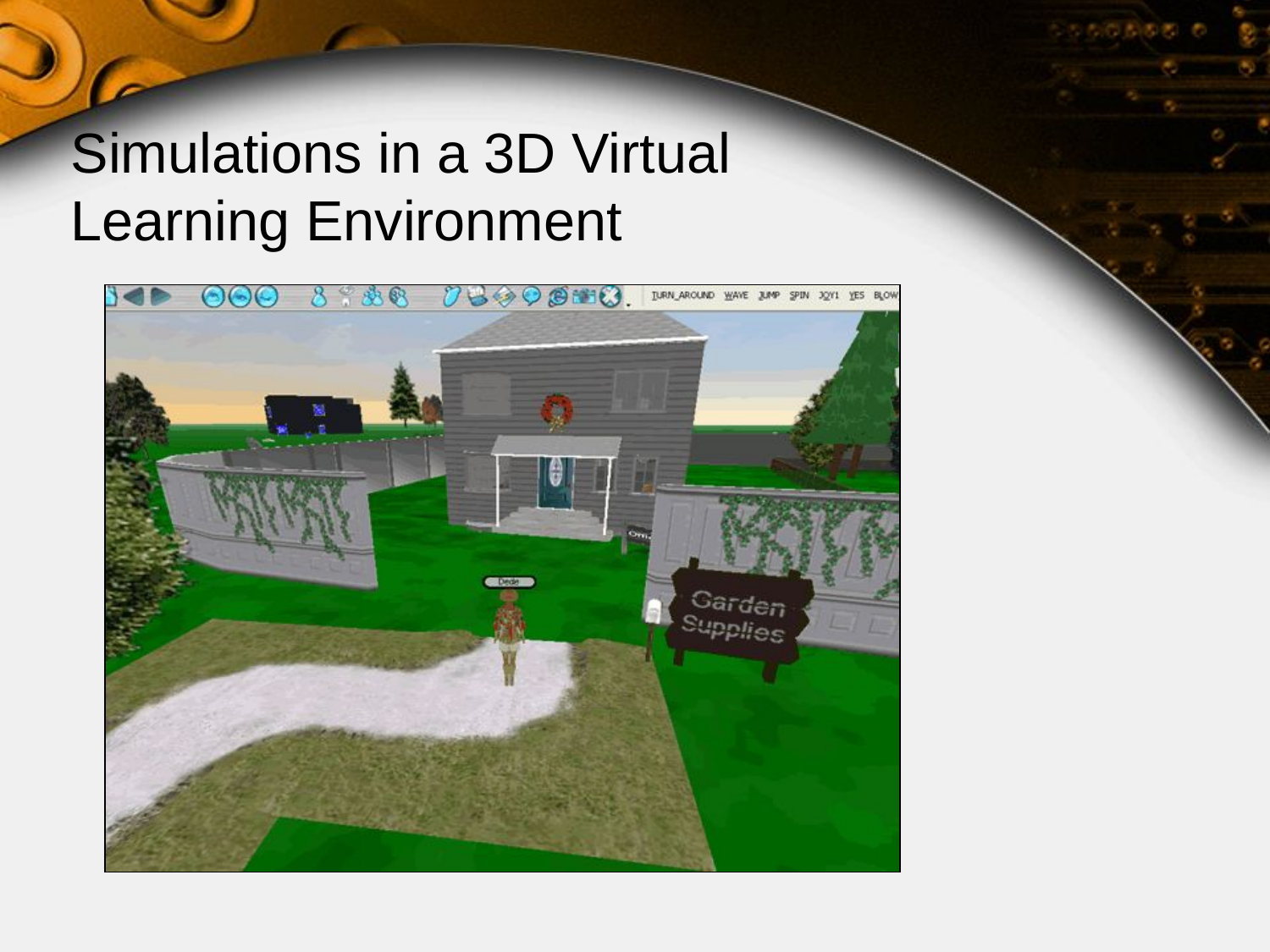

# Simulations in a 3D Virtual Learning Environment

## Slide 20
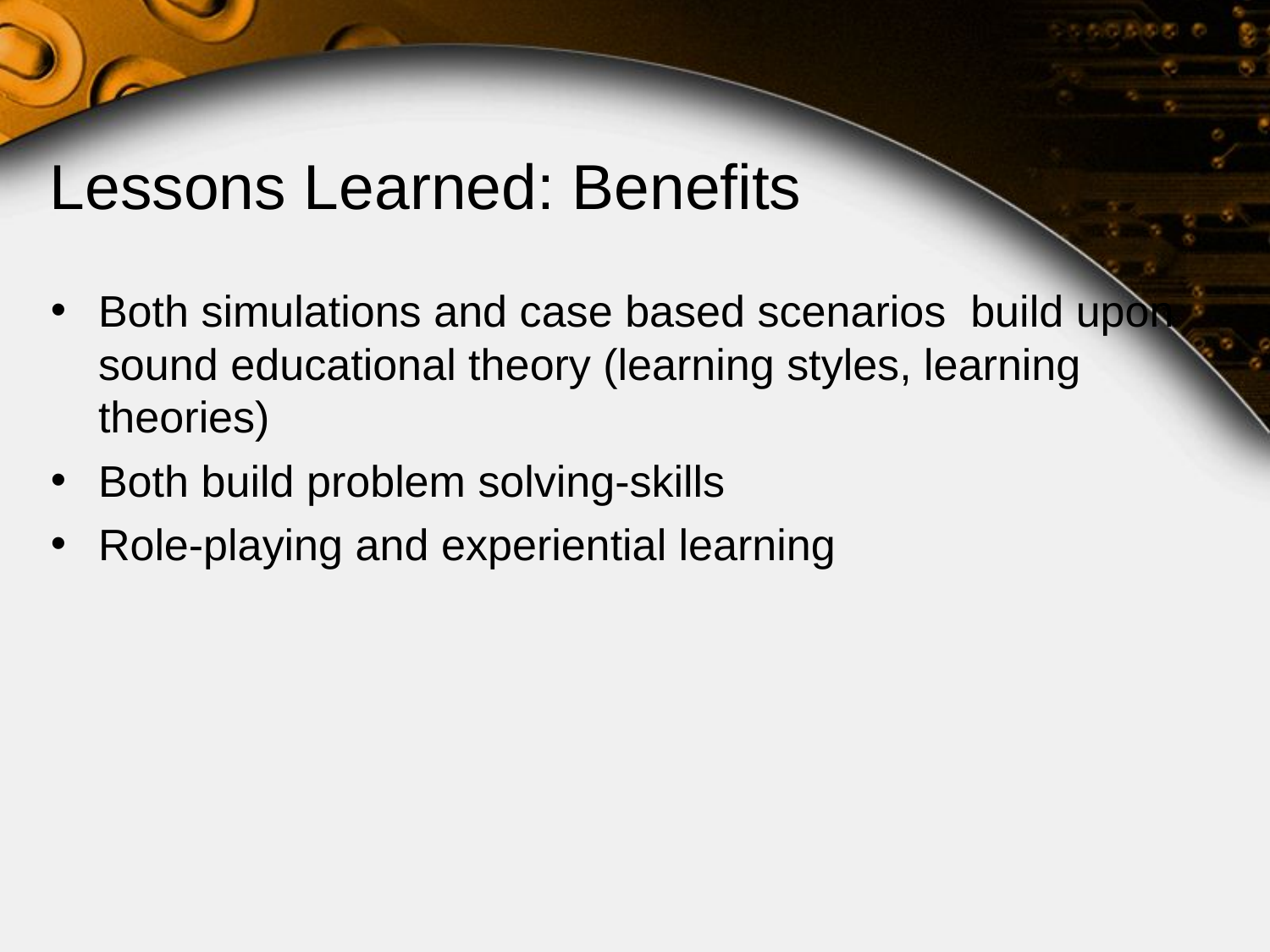

# Lessons Learned: Benefits
Both simulations and case based scenarios build upon sound educational theory (learning styles, learning theories)
Both build problem solving-skills
Role-playing and experiential learning

## Slide 21
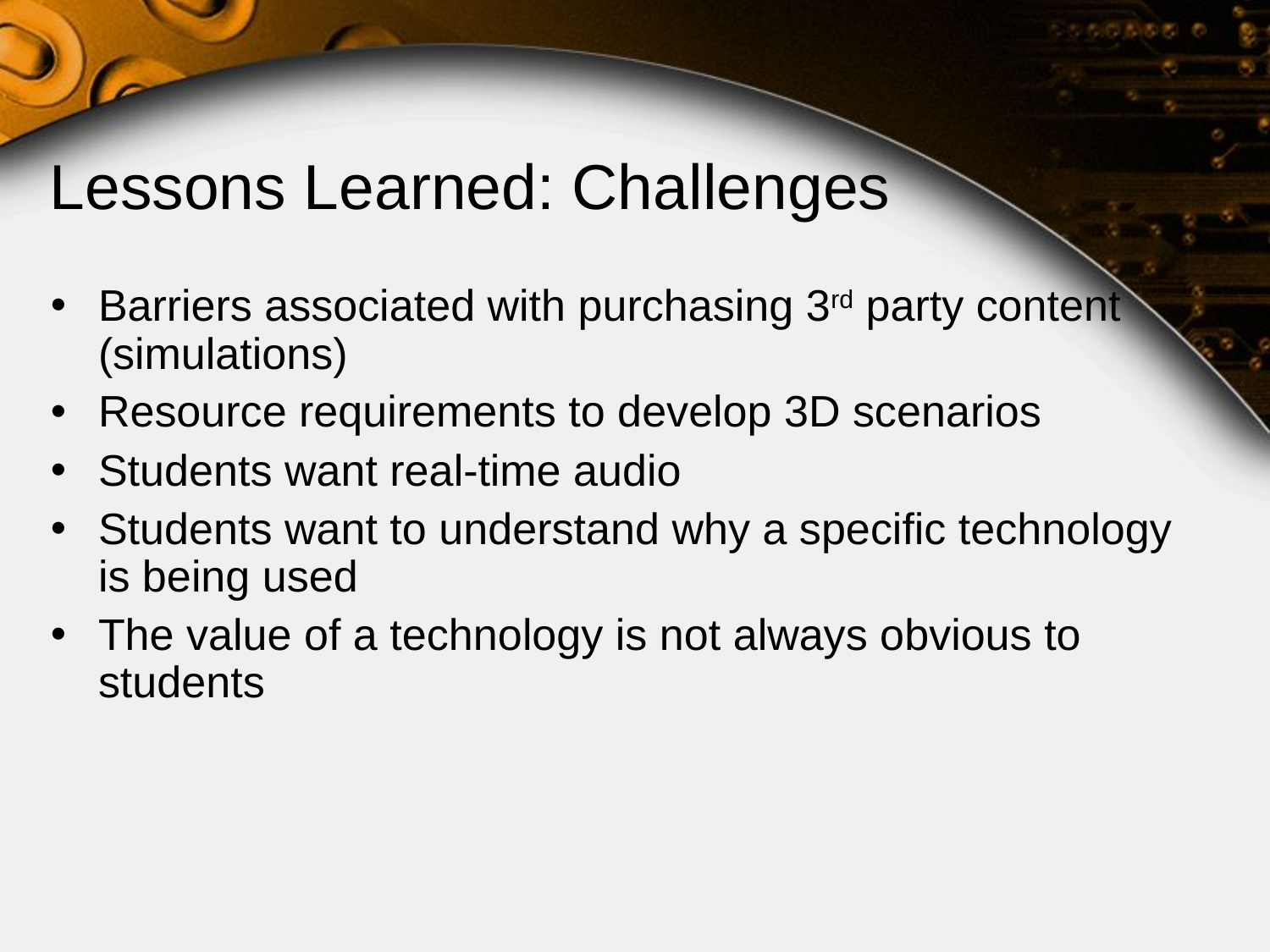

# Lessons Learned: Challenges
Barriers associated with purchasing 3rd party content (simulations)
Resource requirements to develop 3D scenarios
Students want real-time audio
Students want to understand why a specific technology is being used
The value of a technology is not always obvious to students

## Slide 22
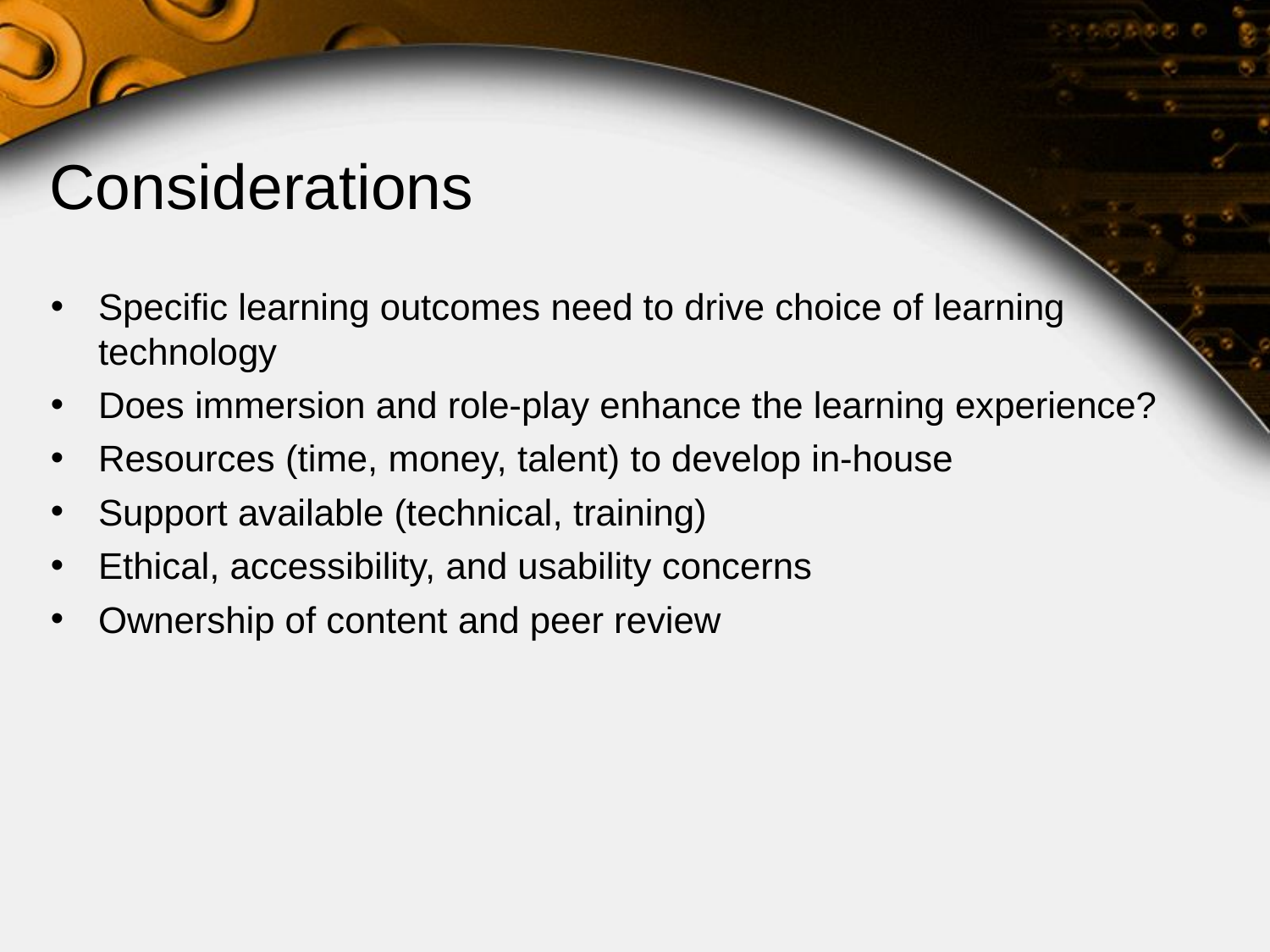

# Considerations
Specific learning outcomes need to drive choice of learning technology
Does immersion and role-play enhance the learning experience?
Resources (time, money, talent) to develop in-house
Support available (technical, training)
Ethical, accessibility, and usability concerns
Ownership of content and peer review
